# Circular RNAs in Alzheimer’s Disease: Mechanisms, Biomarkers, and EPAC Signalling in Ferroptosis

**DOI:** 10.3390/antiox15101321

**Published:** 2026-10-13

**Authors:** Mert Yüksel, Martina Schmidt

**Affiliations:** 1 Department of Molecular Pharmacology, University of Groningen, 9713 AV Groningen, The Netherlands; mert.yuksel20@ogr.atauni.edu.tr; 2 Faculty of Medicine, Atatürk University, Erzurum 25240, Türkiye; 3 Groningen Research Institute for Asthma and COPD (GRIAC), University Medical Center Groningen, University of Groningen, 9713 AV Groningen, The Netherlands

**Keywords:** Alzheimer’s disease, circular RNA, neuroinflammation, tau protein, Amyloid-beta plaques, EPAC, ferroptosis

## Abstract

Alzheimer’s disease (AD) is a progressive neurodegenerative disorder characterized by cognitive decline and complex pathogenesis, involving amyloid-β (Aβ) accumulation, tau pathology, and neuroinflammation, driven by microglial shifts toward pro-inflammatory M1-like states, causing neuronal dysfunction and death, particularly by ferroptosis. Current diagnostic and therapeutic strategies remain limited, highlighting the need for novel molecular regulators. Circular RNAs (circRNAs) are covalently closed non-coding RNAs (ncRNAs), representing promising regulatory molecules and peripheral biomarkers. Evidence suggests that circRNAs regulate AD-related pathways, through microRNA (miRNA) sponging, including modulation of NF-κB/BACE1-dependent (nuclear-factor-kappa-B/beta-site APP-cleaving enzyme 1) amyloidogenic APP (amyloid precursor protein) processing and through disruption of NMDA (N-methyl-D-aspartate) receptor-dependent synaptic signaling, exemplified by circRIMS2-driven UBE2K (ubiquitin-conjugating enzyme E2K)-mediated GluN2B (glutamate ionotropic receptor NMDA type subunit-2B) degradation. Additionally, circRNAs represent promising non-invasive diagnostic biomarkers. Plasma hsa_circRNA_050263 showed high diagnostic accuracy for early-stage AD. CircRNAs may offer a therapeutic strategy for modulating cell-death pathways, particularly ferroptosis. Selectively targeting EPAC2 (exchange protein directly activated by cAMP2) may preserve EPAC2-associated neuroprotection, while limiting EPAC1-associated ferroptosis, although challenges such as blood–brain barrier delivery remain to be addressed. This review summarizes circRNA biogenesis, diagnostic potential, and roles in AD pathogenesis, and limitations and AD models, besides investigating the selective circRNA–EPAC axis.

## 1. Introduction

Alzheimer’s disease (AD) is the most prevalent form of dementia and a leading cause of disability and death in older adults worldwide [1]. In 2025, 56.9 million people were living with a diagnosis of dementia, and this number is expected to exceed 152.8 million by 2050 [1]. Clinically, AD is characterised by a gradual decline in episodic memory and executive function and, ultimately, by loss of the capacity for independent living, imposing a substantial medical, economic and societal burden [2]. This burden is not distributed equally between the sexes [3]. Women account for nearly two-thirds of all AD cases worldwide and show faster cognitive decline after diagnosis [3]. The higher prevalence of AD in women has increased interest in the role of biological sex in AD pathogenesis. This may also extend to circRNA regulation, as circHOMER1 is downregulated in the entorhinal cortex specifically in females with AD [4]. Despite extensive research, clinical diagnosis continues to rely heavily on cognitive testing and structural imaging [5], even though molecular changes precede clinical presentation by many years, with Aβ abnormalities detectable approximately 15–20 years before diagnosis and tau pathology and neurodegenerative changes developing subsequently [5]. The recently developed disease-modifying anti-amyloid monoclonal antibodies (aducanumab, lecanemab, donanemab) provide measurable but modest benefit limited largely to the earliest disease stages of disease [6]. Together, the need for earlier diagnosis and more effective disease-modifying interventions supports the investigation of molecular markers capable of detecting early disease and molecular targets capable of modifying its progression.

The neuropathological features of AD are extracellular plaques containing aggregated Aβ (amyloid-β), generated through sequential β- and γ-secretase cleavage of APP (amyloid precursor protein), and intraneuronal neurofibrillary tangles composed of hyperphosphorylated tau [7,8]. Tau hyperphosphorylation disrupts its normal association with microtubules, contributing to cytoskeletal instability and impaired axonal transport [9]. These pathological changes are accompanied by chronic neuroinflammation involving multiple glial cell types, particularly microglia and astrocytes [10]. Microglia, the resident immune cells of the central nervous system, can shift from a homeostatic, M2-like phenotype toward a pro-inflammatory M1-like state, characterized by increased cytokine production and oxidative stress [11,12], although the conventional M1/M2 classification does not fully represent the heterogeneity of microglial states observed in AD [13]. Similarly, astrocytes, the most abundant glial cells in the central nervous system, contribute to Aβ clearance, antioxidant defence, and brain iron homeostasis and may transition from a homeostatic A2-like state toward a reactive, neurotoxic A1-like phenotype, with impaired homeostatic functions and increased inflammatory activity [14], which is not discussed in the context of circRNA (circular RNA) and AD. These glial alterations may contribute to neuronal injury and regulated cell-death pathways, including ferroptosis, which is characterized by iron-dependent lipid peroxidation and oxidative membrane damage [15,16,17].

Clinically, these mechanistic complexities are further reflected in the substantial overlap between AD and other dementias, including dementia with Lewy bodies, vascular dementia, and Parkinson’s disease dementia [18]. Although these disorders share features such as synaptic dysfunction and progressive cognitive decline, they differ in their underlying molecular mechanisms and therapeutic responses [18]. Distinguishing AD from other forms of dementia remains an important diagnostic challenge, and circRNAs have emerged as potential biomarkers for differential diagnosis [18,19]. Accordingly, this review focuses on circRNA-mediated mechanisms and biomarker development in AD, including their potential relevance to distinguishing AD from non-AD dementias, while considering the broader dementia spectrum where appropriate.

ncRNAs (noncoding RNAs) have emerged as novel post-transcriptional regulators of neuronal physiology implicated in AD pathogenesis, and circRNAs represent a distinct subclass of ncRNAs [20,21]. Generated by back-splicing of pre-mRNAs (messenger RNAs), circRNAs are covalently closed single-stranded molecules that lack 5′ caps and 3′ poly-A tails [22,23]. This topology makes them highly resistant to exonuclease-mediated degradation and yields a half-life that substantially exceeds that of their linear mRNA counterparts, conferring exceptional stability in plasma, serum, exosomes, and cerebrospinal fluid [22,23]. Each circRNA carries a unique BSJ (back-splice junction), which provides the principal sequence feature for its specific detection and manipulation relative to its linear cognate [24]. CircRNAs are highly abundant in the central nervous system, often exhibit brain-region– and cell-type–specific expression, and regulate gene-expression programs through several distinct mechanisms: competing with linear mRNAs for miRNA (microRNA) binding, sequestering or scaffolding RBPs (RNA-binding proteins), modulating transcription of their own parental loci, and—in a small but growing subset of cases—templating cap-independent translation of functional or pathogenic peptides [20,21,22].

Several AD-associated circRNAs illustrate this functional diversity. ciRS-7 (CDR1as), a highly abundant brain-enriched circRNA, has been linked to Aβ homeostasis through miR-7/UBE2A (Ubiquitin Conjugating Enzyme E2 A)-dependent Aβ clearance and NF-κB/UCHL1 (Nuclear Factor kappa-light-chain-enhancer of activated B cells/Ubiquitin C-terminal hydrolase L1) -dependent regulation of APP and BACE1 (Beta-site Amyloid precursor protein Cleaving Enzyme 1) degradation [25,26]. circCwc27, which is increased in AD mouse models and human post-mortem brain tissue, interacts with Pur-α and alters APP transcription and neprilysin-mediated Aβ clearance [27]. A different mechanism is represented by circMAN2A1, in which adenosine-to-inosine (A to I) editing alters its coding potential and is associated with translation of chimeric peptides whose abundance increases with progressive AD pathology [28]. These examples illustrate that circRNAs can influence AD-related processes through miRNA-dependent and protein-mediated regulation as well as through their own coding potential, while their stability in biofluids also supports their investigation as candidate biomarkers.

Recent evidence has extended circRNA involvement across several AD-related processes. On the amyloidogenic axis, loss of circHDAC9 shifts APP processing towards BACE1 through the miR-138/SIRT-1/ADAM10 (Sirtuin 1/A disintegrin and metalloprotease 10) pathway [29], whereas circ_0004381 increases expression of the PSEN1 (γ-secretase catalytic subunit presenilin 1) [30]. On the synaptic axis, circRIMS2 promotes UBE2K (Ubiquitin Conjugating Enzyme E2 K)-dependent degradation of the NMDA (N-methyl-D-aspartate) receptor subunit GluN2B (glutamate ionotropic receptor NMDA type subunit-2B), with associated reductions in dendritic spine density and memory performance [31], while circ-Bptf has been linked to synaptic integrity through the miR-138-5p/p62 pathway [32]. Neuroinflammatory regulation is represented by circAPP, which promotes a pro-inflammatory microglial phenotype through the miR-1906/CLIC1 (Chloride intracellular channel protein 1) axis [33], and circ_0061183, whose reduction is associated with increased pro-inflammatory microglial activation [34]. circDlg1 additionally links circRNA regulation to second messenger signalling through the PDE4B/cAMP/PKA/CREB (Phosphodiesterase 4B/Cyclic adenosine monophosphate/Protein kinase A/cAMP response element-binding protein) pathway [35]. Preclinical studies further indicate that either suppression of pathogenic circRNAs or restoration of protective circRNAs can improve molecular and behavioural outcomes in AD models. Sex-specific regulation may also be relevant, as circHOMER1 is downregulated in the entorhinal cortex in a female-specific manner [4].

CircRNAs have also been investigated as circulating biomarkers (Table 1). In plasma, hsa_circRNA_050263—predicted to interact with the ATP13A1 (ATPase class VI type 13A1)—discriminates AD and mild cognitive impairment from healthy controls with an area under the curve of 0.98 [36], while hsa_circRNA_100837 from the same cohort shows statistically significant overexpression predominantly in female AD participants, reflecting the established female predisposition to the disease and reinforcing the need for gender-stratified biomarker development [36] (Table 1). However, the current biomarker evidence derives from a limited number of relatively small cohorts, and most candidates lack independent replication or direct comparison with established plasma biomarkers such as p-tau217 (phosphorylated tau 217) in the same participants [37].

Despite the increasing number of reported AD-associated circRNAs, several important gaps remain. Mechanistic studies rely predominantly on neuroblastoma-derived and other immortalised cell lines and amyloid-driven transgenic models, and circRNAs investigated as diagnostic candidates are largely distinct from those characterised mechanistically. The relationship with tau remains particularly underexplored: no AD-associated circRNA has yet been shown to directly regulate tau, despite the importance of tau to both neurofibrillary pathology and APP-dependent neuronal iron export. Similarly, although ferroptosis is increasingly implicated in AD, direct circRNA regulation of ferroptosis has not been demonstrated in an AD model, and evidence that circRNAs can regulate ferroptosis currently derives from other disease contexts. Commonly measured outcomes in AD circRNA studies, including apoptosis, general oxidative stress and inflammatory markers, are insufficient by themselves to establish ferroptosis. CircRNA functions in astrocytes also remain poorly characterised. These limitations are compounded by the experimental models used to study a disease that develops over decades and involves interactions among neurons, microglia and astrocytes. Human iPSC (Induced pluripotent stem cell)-derived co-cultures and organoid systems may address some of these limitations, although these models also require further refinement [38]. Therapeutic translation presents additional challenges, including BSJ-specific manipulation, context-dependent circRNA functions and efficient delivery to the central nervous system.

Building on these advances, the present review provides an integrated, mechanism-driven synthesis of the current evidence on circRNAs in AD. We first outline the biogenesis and molecular functions that distinguish circRNAs from other RNAs. We then examine the diagnostic potential of plasma- and CSF (cerebrospinal fluid)-based circRNA biomarkers (Table 1), followed by AD-associated circRNAs involved in amyloidogenic processing, synaptic dysfunction and neuroinflammation (Table 2). Ferroptosis is subsequently examined through its three major components—iron homeostasis, lipid metabolism and antioxidant defence—and their relationships with Aβ, tau pathology and neuroinflammation, as well as interaction with circRNAs. Within this context, we examine cAMP–EPAC (exchange protein directly activated by cAMP) signalling as a potential interface between circRNA regulation, synaptic dysfunction and ferroptosis, with particular attention to the distinct roles of EPAC1 (1) and EPAC2 (2). Finally, we discuss the current limitations of circRNA biomarkers and therapeutic approaches, the evidence gaps surrounding circRNA–tau and circRNA–ferroptosis relationships, and experimental models that may help address these questions. Together, this review aims to distinguish established circRNA mechanisms from emerging hypotheses and to identify experimentally testable directions for circRNA research in AD.

## 2. Ferroptosis and Alzheimer’s Disease

Ferroptosis was first described by Dixon as an iron-dependent, non-apoptotic form of regulated cell death characterized by the accumulation of lipid peroxides and distinct mitochondrial morphological changes, including mitochondrial shrinkage and increased membrane density [16]. It is biochemically and genetically distinct from apoptosis, necroptosis, and autophagy-dependent cell death, and can be suppressed by iron chelation and lipophilic radical-trapping antioxidants rather than by caspase inhibition [16,39]. Increasing evidence suggests that ferroptosis can be a central event in AD. Ferroptosis involves three closely interconnected processes in AD: iron homeostasis, lipid metabolism, and antioxidant defence system [39].

### 2.1. Iron Homeostasis and Alzheimer’s Disease

Higher brain iron levels are associated with faster cognitive decline, whereas this association is absent in individuals without AD pathology, suggesting an interaction between iron accumulation and AD-related proteinopathies rather than the effect of aging alone [40].

**Figure 1:**
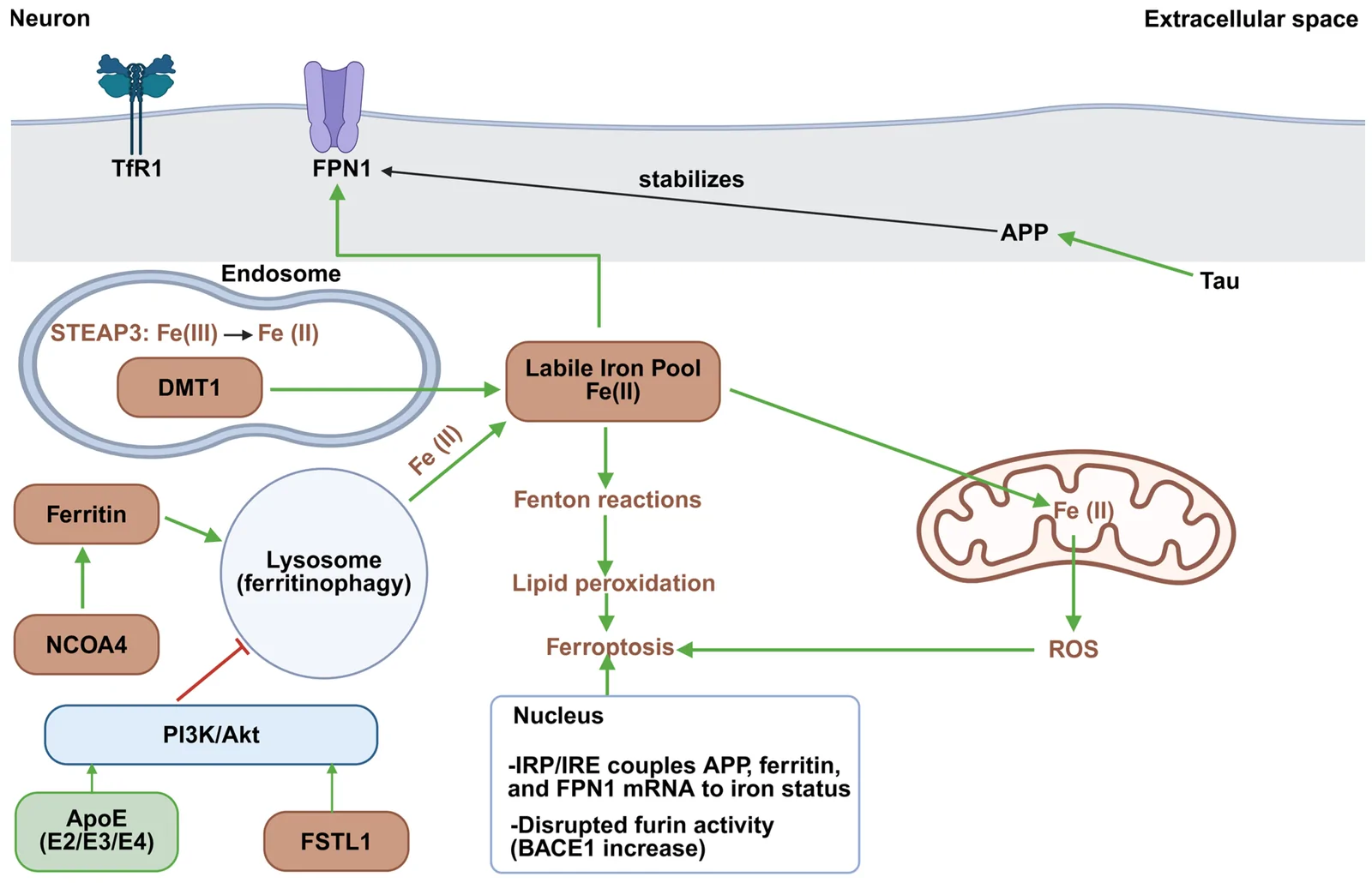
Regulation of neuronal iron homeostasis and ferroptosis through iron trafficking, ferritinophagy, and PI3K/Akt signalling (Created in BioRender. Yuksel, M. (2026). https://BioRender.com/pk9ccjs). Green arrows indicate directional activation, transport, or progression between molecular processes. The black arrow indicates APP-mediated stabilisation of FPN1 at the neuronal membrane. The red blunt-ended line indicates inhibition of ferritinophagy by PI3K/Akt signalling. TfR1-mediated uptake delivers transferrin-bound Fe(III) to endosomes, where STEAP3 reduces Fe(III) to Fe(II), followed by DMT1-mediated release into the cytosolic LIP [41,42,43]. Ferritin sequesters intracellular iron, whereas NCOA4-mediated ferritinophagy promotes lysosomal ferritin degradation and releases Fe(II) into the LIP [44,45,46]. Excess labile Fe(II) promotes Fenton reactions and lipid peroxidation, thereby increasing ferroptotic susceptibility [16,39]. A fraction of intracellular Fe(II) also enters mitochondria, where altered iron handling can increase ROS generation and contribute to ferroptotic injury [41,42,43]. Neuronal iron export is mediated by FPN1 [47]. Tau facilitates APP trafficking to the neuronal membrane, where APP stabilises FPN1 and supports iron efflux [48,49]. Loss of soluble tau may therefore impair APP-dependent FPN1-mediated iron export and promote neuronal iron retention [49]. ApoE activates PI3K/Akt signalling, which suppresses ferritinophagy and limits ferritin-derived iron release [46]. In Aβ-treated neuronal cells, increased FSTL1 is associated with reduced PI3K/Akt activity, whereas FSTL1 silencing restores PI3K/Akt signalling and attenuates ferroptosis-associated changes [56,57]. The IRP/IRE system couples cellular iron availability to the expression of iron-related proteins, including APP, ferritin, and FPN1, while altered iron homeostasis may impair furin activity and increase BACE1 expression, providing an additional link between iron dysregulation, amyloidogenic APP processing, and ferroptotic susceptibility [54].

Neuronal iron uptake is mediated principally by TfR1 (transferrin receptor 1), which binds transferrin-bound Fe^3+^ [41]. Then, the complex is endocytosed, and iron is released in endosomes, reduced to Fe^2+^ by STEAP3 (Six-transmembrane epithelial antigen of the prostate 3) and transported into the cytoplasm through DMT1 (Divalent metal transporter 1) [42,43]. Cytosolic iron enters the LIP (Labile iron pool), a redox-active iron fraction capable of catalysing Fenton reactions [44]. The LIP is regulated not only by iron uptake but also by intracellular storage and release [45]. NCOA4 (Nuclear receptor coactivator 4)-mediated ferritinophagy targets ferritin for lysosomal degradation, releasing stored iron into the LIP and thereby sensitizing the cell to ferroptosis [46]. A fraction of the intracellular iron pool is also transported into mitochondria for heme and iron–sulfur cluster synthesis, and disruption of mitochondrial iron handling can increase oxidative stress and ferroptotic susceptibility (Figure 1).

Efflux depends almost entirely on FPN1 (Ferroportin 1), the only known vertebrate iron exporter [47]. This process is closely linked to AD biology. APP stabilizes FPN1 at the neuronal surface and thereby facilitates iron efflux, whereas tau promotes the trafficking of APP to the membrane [48,49]. Consequently, reduced soluble tau suppresses FPN1 activity, resulting in neuronal iron retention [49]. Aging, chronic inflammation and oxidative stress may further disrupt iron export and promote intracellular iron accumulation [50,51] (Figure 1).

Iron dysregulation is closely associated with both Aβ and tau pathology. Iron accumulation promotes tau hyperphosphorylation through activation of CDK5 (Cyclin-dependent kinase 5) and GSK-3β (glycogen synthase kinase-3β), thereby facilitating neurofibrillary tangle formation [52]. HO-1 (Heme oxygenase-1) is also present within neurofibrillary tangles, and increased HO-1 expression has been associated with iron accumulation and enhanced tau phosphorylation [53]. Iron overload can also alter the expression of APP, ferritin, and FPN1 through IRP/IRE (iron-regulatory protein/iron-responsive element) interactions and disrupt furin activity, resulting in BACE1 upregulation [54]. Aβ pathology can further impair cellular iron homeostasis, potentially increasing iron accumulation and oxidative stress [55]. Beyond its interactions with Aβ and tau pathology, excess redox-active iron promotes lipid peroxidation through Fenton chemistry, thereby increasing neuronal susceptibility to ferroptosis [16,39] (Figure 1).

### 2.2. Lipid Metabolism and Alzheimer’s Disease

**Figure 2:**
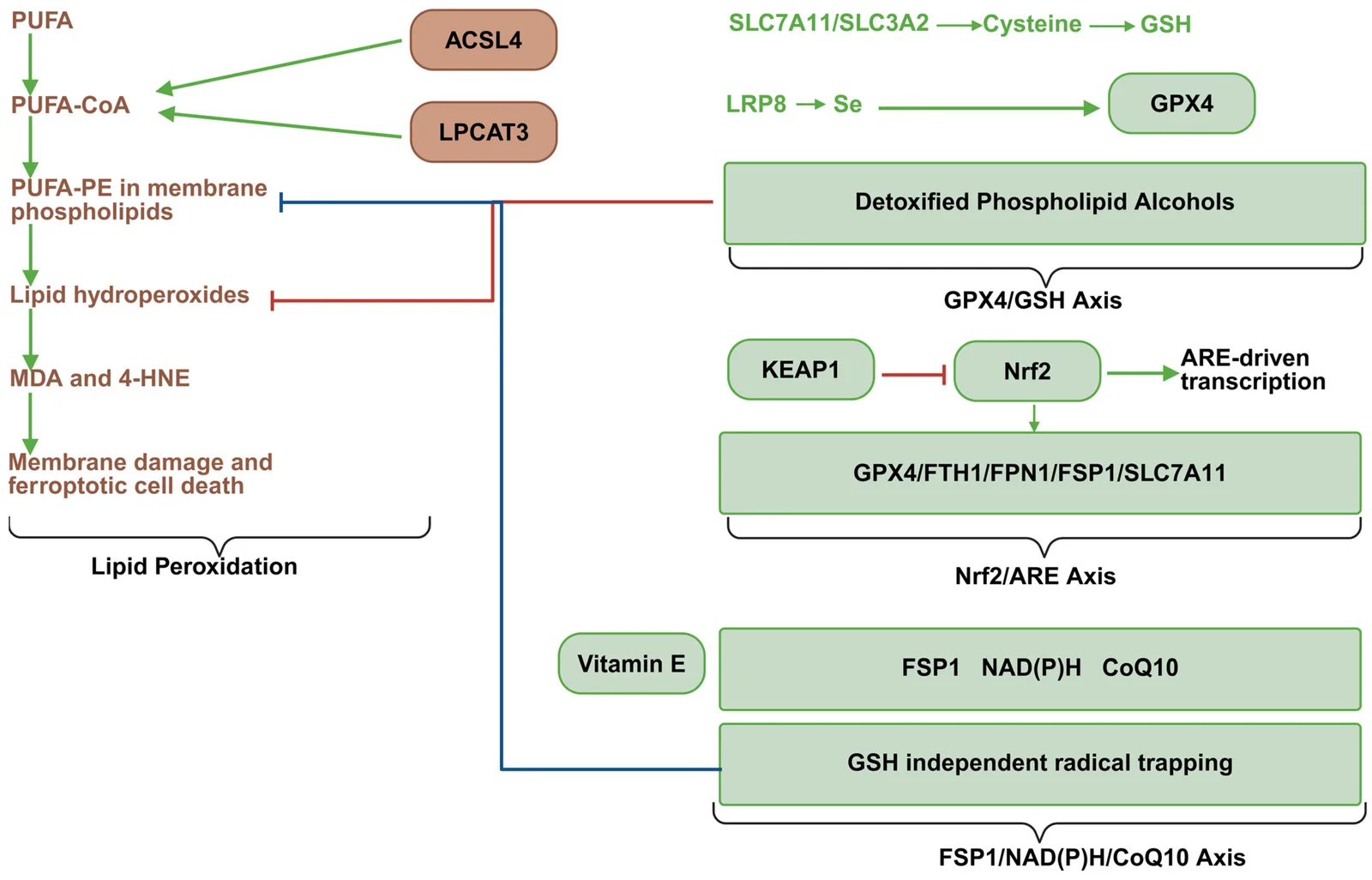
Lipid peroxidation and major antioxidant defence pathways regulating ferroptosis (Created in BioRender. Yuksel, M. (2026) https://BioRender.com/hu3y5xd). Green arrows indicate directional activation, enzymatic conversion, or pathway progression. Red blunt-ended lines indicate inhibitory effects. PUFAs are converted to PUFA-CoA and incorporated into membrane phospholipids as PUFA-PE, processes facilitated by ACSL4 and LPCAT3 [63,64]. Oxidation of PUFA-containing phospholipids generates lipid hydroperoxides and secondary lipid-peroxidation products, including MDA and 4-HNE, ultimately promoting membrane damage and ferroptotic cell death [63,64]. This process is opposed by several antioxidant systems [59]. GPX4/GSH axis reduces phospholipid hydroperoxides to non-toxic phospholipid alcohols. GSH synthesis depends on cystine uptake through the antiporter system, comprising SLC7A11 and SLC3A2, whereas selenium availability through the LRP8 pathway supports GPX4 synthesis and activity [65]. The Nrf2/ARE pathway provides an additional protective mechanism: release of Nrf2 from KEAP1-mediated repression promotes ARE-dependent transcription of ferroptosis-related antioxidant and iron-homeostatic genes, including GPX4, FTH1, FPN1, FSP1, and SLC7A11 [66,67,68]. In parallel, the FSP1/NAD(P)H/CoQ10 system suppresses lipid peroxidation independently of GSH by regenerating reduced CoQ10 as a radical-trapping antioxidant, with vitamin E providing additional lipid-phase antioxidant protection [69,70]. Together, these pathways determine the balance between membrane lipid peroxidation and antioxidant defence that regulates ferroptotic susceptibility.

Lipid peroxidation is a central process in ferroptotic cell death and is increased in AD [58]. In the 5xFAD model, elevated levels of the lipid peroxidation products MDA (malondialdehyde) and 4-HNE (4-hydroxynonenal) are accompanied by alterations in membrane phospholipid metabolism [59]. Increased MDA and 4-HNE levels have also been reported in brain tissue, CSF, and blood from patients with AD [60]. Importantly, lipid peroxidation products can directly influence AD-related molecular processes [61]. For example, 4-HNE modifies nicastrin, a component of the γ-secretase complex, increasing its interaction with the APP-C99 fragment and thereby enhancing γ-secretase activity and Aβ42 production [62]. This modification has also been detected in human AD brain and is associated with increased γ-secretase activity and Aβ plaque burden [62]. These findings provide a mechanistic link between lipid peroxidation and amyloidogenic APP processing in AD (Figure 2).

Ferroptosis depends on PUFAs (polyunsaturated fatty acids) incorporated into membrane phospholipids rather than on free PUFAs [62]. Phospholipids containing arachidonic acid and adrenic acid, particularly phosphatidylethanolamines, are highly susceptible to oxidation because of the multiple double bonds within their fatty acid chains, thereby promoting lipid peroxidation [62]. Two enzymes are particularly important in this process: ACSL4 (acyl-CoA synthetase long-chain family member 4) converts long-chain PUFAs into their acyl-CoA derivatives, while LPCAT3 (lysophosphatidylcholine acyltransferase 3) facilitates their incorporation into membrane phospholipids [63]. Loss of ACSL4 alters membrane lipid composition and reduces susceptibility to ferroptosis, establishing ACSL4 as an important regulator of this process [62]. This mechanism may be particularly relevant in the brain because of its high abundance of long-chain PUFAs. Consistent with this, inhibition of ACSL4-mediated lipid metabolism, including mitochondrial aldehyde dehydrogenase activity or suppression of the AMPK/Sp1/ACSL4 (Adenosine Monophosphate-Activated Protein Kinase/Specificity Protein 1) pathway, reduces neuronal ferroptosis in APP/PS1 models [71,72] (Figure 2).

Lipoprotein metabolism may provide an additional link between lipid homeostasis and ferroptotic susceptibility in AD. ApoE has been shown to suppress ferroptosis by activating PI3K/Akt signalling and inhibiting ferritinophagy, thereby limiting the release of ferritin-bound iron and subsequent lipid peroxidation [56]. In the same study, ApoE2, ApoE3 and ApoE4 showed comparable protection against erastin-induced ferroptosis in vitro, indicating that the increased susceptibility associated with APOE ε4 cannot be explained by lower intrinsic anti-ferroptotic activity of the ApoE4 isoform [71]. Instead, lower ApoE abundance and differences in lipid composition in APOE ε4 carriers have been proposed as potential contributors to increased ferroptotic susceptibility [56].

### 2.3. Antioxidant Defence System and Alzheimer’s Disease

Neurons are susceptible to oxidative damage because of their high PUFA content, high oxygen consumption and relatively limited antioxidant capacity [73]. In AD, alterations in major ferroptosis-protective systems, including GPX4/GSH (Glutathione peroxidase 4/glutathione), Nrf2/ARE (Nuclear factor erythroid 2-related factor 2/Antioxidant response element) and FSP1/NAD(P)H/CoQ10 (Ferroptosis Suppressor Protein 1/Nicotinamide Adenine Dinucleotide Phosphate/Coenzyme Q10), can reduce cellular defence against iron-dependent lipid peroxidation [59,69,74]. Ferroptosis-related oxidative damage may also interact with Aβ and tau pathology, with each process potentially contributing to the progression of the others.

#### 2.3.1. The GPX4/GSH Axis

The GPX4/GSH system is a major cellular defence against ferroptosis and is particularly important in neurons, where high membrane PUFA content increases susceptibility to lipid peroxidation [75]. GPX4 uses GSH to reduce phospholipid hydroperoxides and thereby protects neuronal membranes from ferroptotic damage [75]. The importance of GPX4 for neuronal survival has been demonstrated experimentally: conditional deletion of GPX4 in adult neurons markedly increased lipid peroxidation and caused progressive neuronal degeneration, demonstrating that loss of GPX4-mediated control of membrane lipid oxidation is sufficient to compromise neuronal survival [76,77]. GSH synthesis depends on an adequate supply of cysteine, which is partly provided through cystine uptake by the cystine/glutamate antiporter system, composed of SLC7A11 (Solute Carrier Family 7A11) and SLC3A2 (3A2) [16].

Impairment of the GPX4/GSH system has also been associated with Aβ-induced neuronal injury [78]. For example, injection of Aβ25–35 into the rat entorhinal cortex decreased SLC7A11 and GPX4 expression and increased transferrin receptor expression, accompanied by neuronal damage and impaired spatial learning and memory [78]. Treatment with ferrostatin-1 attenuated these pathological and behavioural changes, supporting the involvement of ferroptosis in Aβ-induced neurotoxicity [78]. These findings indicate that Aβ pathology is associated with impairment of the SLC7A11/GSH/GPX4 defence system and increased neuronal susceptibility to ferroptosis [78].

PSEN mutations provide further evidence linking ferroptosis to AD. γ-Secretase-dependent Notch-1 signalling promotes the expression of LRP8 (low-density lipoprotein receptor-related protein 8), which mediates the neuronal uptake of selenoprotein P and supports selenium availability for GPX4 synthesis [65]. PSEN mutations impair this pathway, resulting in reduced selenium uptake and GPX4 expression and increased susceptibility to ferroptosis [65]. This finding indicates that PSEN1 dysfunction may influence neuronal survival through ferroptosis in addition to its established effects on APP processing [65] (Figure 2).

The GPX4/GSH pathway may therefore represent a therapeutic target in AD. Experimental restoration of GSH availability or GPX4 activity reduces neuronal ferroptosis and improves cognitive outcomes in AD models [79,80]. Ganoderic acid A has been reported to reduce ferroptosis through Nrf2/SLC7A11/GPX4 signalling, while tetrahydroxy stilbene glycoside modulates GSH/GPX4/ROS signalling in APP/PS1 mice [79,80]. However, because these compounds affect multiple cellular pathways, further studies are required to determine the contribution of ferroptosis inhibition to their neuroprotective effects (Figure 2).

#### 2.3.2. The Nrf2/ARE Axis

Nrf2 is an important transcriptional regulator of the oxidative stress response and iron homeostasis [66,67]. Under basal conditions, Keap1 (Kelch-like ECH-associated protein 1) promotes Nrf2 degradation, whereas oxidative stress stabilizes Nrf2 and promotes its nuclear translocation and transcriptional activity [66,67]. Nrf2 regulates several proteins involved in ferroptosis, including those associated with glutathione metabolism, iron storage and export, and lipid peroxide defence, such as GPX4, FTH1 (ferritin heavy chain 1), FPN1, and FSP1 (ferroptosis suppressor protein 1) [66,67]. Nrf2 signalling can therefore influence cellular susceptibility to ferroptosis through multiple mechanisms [67] (Figure 2).

Nrf2 function is impaired in AD and has been linked to Aβ-induced ferroptotic neuronal injury [68]. Aβ can impair Nrf2 activity, whereas inhibition of Keap1 restores Nrf2 signalling and reduces Aβ-induced neuronal toxicity [68]. Nrf2 deficiency in APP/PS1 mice also aggravates AD-related pathological changes. Consistent with these findings, pharmacological activation of Nrf2, including through Keap1–Nrf2 interaction inhibitors, eriodictyol, and forsythoside A, has been reported to reduce ferroptosis-associated changes and improve cognitive outcomes in experimental models [68,81,82,83,84] (Figure 2). However, because Nrf2 regulates a broad range of antioxidant, metabolic, and inflammatory processes, the neuroprotective effects of these interventions cannot be attributed exclusively to ferroptosis inhibition.

#### 2.3.3. The FSP1/NAD(P)H/CoQ10 Axis

FSP1 provides a glutathione-independent defence against ferroptosis [69]. FSP1 uses NAD(P)H to maintain CoQ10 in its reduced antioxidant form, which limits lipid peroxidation within cellular membranes [69,70]. This mechanism can protect cells from ferroptosis independently of the GSH/GPX4 system and is also regulated in part by Nrf2 signalling [69,71] (Figure 2).

The role of FSP1 in AD remains poorly characterised. Although alterations in CoQ10 metabolism and oxidative stress have been implicated in neurodegeneration, direct evidence linking FSP1 dysfunction to AD pathology is currently limited [85]. CoQ10 supplementation has been reported to reduce lipid peroxidation and improve functional outcomes in the hippocampus, striatum, and cortex of aged mice [85]; however, these findings do not establish an FSP1-dependent mechanism or demonstrate ferroptosis specifically. Further studies are therefore required to determine whether FSP1-mediated protection against lipid peroxidation is altered in AD and whether modulation of this pathway can reduce neuronal ferroptosis.

## 3. CircRNA Biogenesis

**Figure 3:**
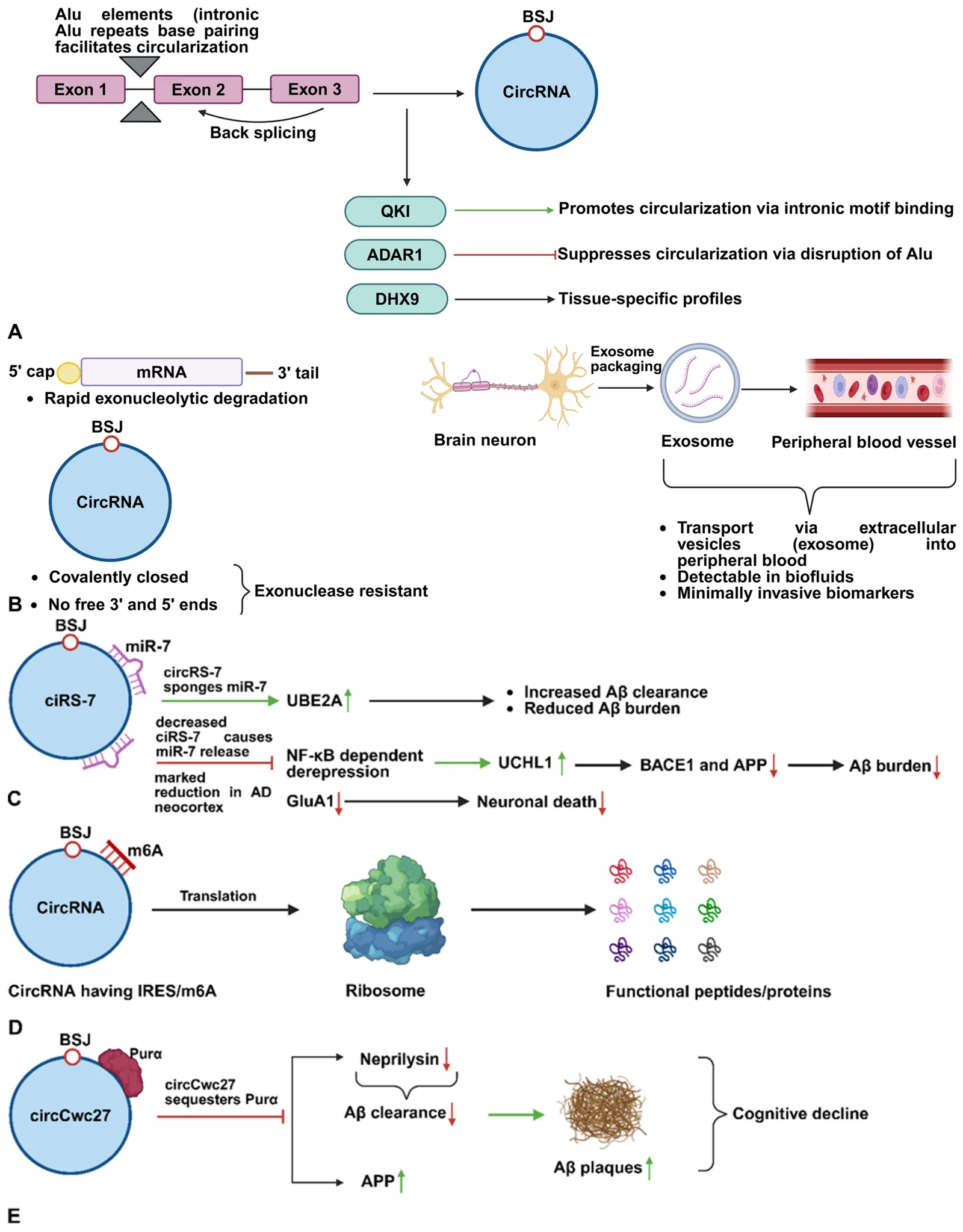
Biogenesis, properties, and molecular functions of circRNA relevant to AD (Created in BioRender. Yuksel, M. (2026) https://BioRender.com/hxqvrg7), (Created in BioRender. Yuksel, M. (2026) https://BioRender.com/agb0g2f). (**A**) Biogenesis and regulation. circRNAs arise from pre-mRNA by back-splicing, in which a downstream splice donor is joined to an upstream acceptor; base-pairing of intronic inverted-repeat (Alu) elements approximates the splice sites, and the covalently closed product carries a unique BSJ. Back-splicing is promoted by QKI (dimerizing on intronic motifs) and suppressed by ADAR1 (disrupting Alu base-pairing), while DHX9 and MBNL shape tissue-specific profiles [24,89,90,91,92]. (**B**) Stability and detection. Lacking free 5′ cap and 3′ poly-A ends, circRNAs resist exonucleolytic degradation and are stable in plasma, serum, CSF, and exosomes, making them attractive biomarkers (Table 1); the BSJ is a unique signature detected by RNA-sequencing algorithms [24]. (**C**) Molecular functions. As miRNA sponges, the brain-enriched circRNA ciRS-7 (also known as CDR1as) contains numerous binding sites for miR-7 [25,26,46]. Reduced ciRS-7 abundance in the AD neocortex may increase the availability of miR-7, resulting in downstream changes in miR-7-regulated pathways. Increased miR-7 availability can suppress UBE2A, a component of the ubiquitin–proteasome system, thereby potentially impairing Aβ clearance [25]. In parallel, reduced ciRS-7-mediated sequestration of miR-7 has been linked to NF-κB-dependent derepression of UCHL1, with downstream effects on APP and BACE1 levels [26]. These alterations may contribute to increased Aβ burden [25,26]. Reduced ciRS-7 has also been associated with decreased glutamate receptor subunit GluA1 expression and neuronal dysfunction, providing an additional potential link between circRNA dysregulation and neuronal loss in AD [95]. Together, these observations illustrate how disease-associated alterations in a single brain-enriched circRNA can influence multiple molecular pathways relevant to Aβ metabolism and neuronal homeostasis. (**D**) CircRNA undergoes cap-independent translation via IRES/m6A elements, and those lacking an in-frame stop codon undergo rolling-circle translation to yield concatemers; such translation is favoured under stress, including hypoxia [94,96]. (**E**) circCwc27 has been implicated in the regulation of amyloid pathology through interaction with the protein Pur-α [27]. By sequestering Pur-α, circCwc27 can influence downstream molecular pathways associated with APP metabolism and Aβ clearance [27]. Reduced neprilysin expression or activity may impair enzymatic degradation of Aβ, while increased APP availability can further promote amyloidogenic processing [27]. The combined effects of impaired Aβ clearance and increased APP abundance may contribute to enhanced Aβ accumulation and plaque formation, ultimately promoting cognitive decline [27]. This mechanism illustrates how circRNAs can regulate AD pathogenesis through direct interactions with RNA-binding or regulatory proteins rather than exclusively through miRNA sequestration.

CircRNAs are a class of covalently closed ncRNAs generated from pre-mRNAs [22] (Figure 3). Unlike linear isoforms, such as mRNAs, they lack a 5′ cap and a 3′ poly-A tail, which confers exceptional resistance to RNase activity [22] (Figure 3). Their exceptional resistance makes them extremely stable in the plasma, making them ideal diagnostic and therapeutic candidates [23] (Table 1) (Figure 3). Despite this diagnostic promise, it is important to emphasize that circRNAs are not yet incorporated into routine clinical practice for AD due to small, single-center cohorts and a lack of large-scale, multicenter validation studies.

Every circRNA carries a unique BSJ, the single point at which a downstream exon is joined to an upstream exon in a configuration that cannot arise from linear splicing [24] (Figure 3). Because the rest of a circRNA’s sequence is identical to that of its linear mRNA cognate, the BSJ is its defining molecular signature—the feature exploited by sequencing-based algorithms to distinguish circular from linear transcripts [24] (Figure 3). Complementing this sequence-level signature, the covalently closed topology itself—lacking the free 5′ cap and 3′ poly-A tail of linear mRNA—renders circRNAs resistant to exonucleolytic degradation and confers the exceptional stability that underlies their value as biofluid biomarkers [22,23,24] (Figure 3). Some circRNAs are enriched in specific tissues or cell types, including the brain, and can be released within extracellular vesicles such as exosomes, providing a potential route for their transport from cells of origin into the peripheral circulation [86]. These properties provide a biological basis for investigating brain-enriched circRNAs as minimally invasive biomarkers of central nervous system-associated molecular alterations (Figure 3) [87]. However, these properties alone cannot establish the cellular or regional origin of a circulating circRNA or demonstrate that its abundance directly reflects a specific pathogenic process in the brain. Establishing such a link will require integrated validation across brain tissue, cell- and region-specific expression profiles, extracellular-vesicle cargo, and matched blood samples from the same disease context.

The circularization process is tightly regulated by cis-regulatory elements, such as intronic Alu repeats, and trans-acting factors, particularly RBPs [88,89] (Figure 3). Inverted repeats within introns facilitate the approximation of the downstream and upstream splice sites [88] (Figure 3). RBPs modulate this process through diverse mechanisms [89]. For instance, the QKI (quaking) protein dimerizes by binding to specific intronic motifs and promotes circularization [89] (Figure 3). In contrast, ADAR1 (Adenosine deaminase acting on RNA 1) can suppress circularization by disrupting the base-pairing of intronic Alu elements [90] (Figure 3). Furthermore, proteins like DHX9 (DExH-box helicase 9) and MBNL (muscleblind-like protein) contribute to determining tissue-specific circRNA expression profiles [91,92] (Figure 3).

CircRNAs are involved in regulating numerous biological processes through diverse molecular functions (Figure 3) [22]. The most well-characterized function is miRNA sponging, in which circRNA sequesters specific miRNAs and relieves their suppressive effects on target mRNAs, clarifying the miRNAs’ tissue-specific functions [24] (Figure 3). For instance, while miR-7-5p acts as a direct repressor of targets like EPAC1 in non-neuronal tissues, its activity within the central nervous system is strictly gated by the ciRS-7/Cyrano network to prevent aberrant silencing of synaptic markers [93,94]. In ischemic conditions, downregulation of ciRS-7 releases miR-7, which suppresses AMPA (α-amino-3-hydroxy-5-methyl-4-isoxazolepropionic acid) receptor subunit GluA1 (Gria1), thereby attenuating excitotoxic glutamatergic transmission and reducing neuronal death [95] (Figure 3). CircRNAs also act as protein scaffolds and decoys [24] (Figure 3). They can bind multiple proteins simultaneously, thereby facilitating specific enzyme-substrate interactions [24]. In the context of AD, this protein-binding capacity has been implicated in disease progression. For instance, circCwc27 acts as a protein decoy by directly binding to the RBP Pur-α and trapping it in the cytoplasm [27]. This prevents Pur-α from regulating the transcription of AD-associated genes, including APP, thereby increasing APP expression and Aβ accumulation and contributing to cognitive decline [27] (Figure 3). Similarly, circFOXO3 has been implicated in neuronal apoptosis under glutamate-induced oxidative injury, a process relevant to the synaptic dysfunction and neuronal loss in AD [93]. In hippocampal HT22 neurons, glutamate exposure increases ROS (reactive oxygen species), upregulates circFOXO3, and promotes its interaction with the pro-apoptotic BimEL, accompanied by mitochondrial membrane depolarization, increased cytochrome c release and elevated cleaved caspase-3 expression. These effects are reversed by circFOXO3 silencing [93]. CircFOXO3 knockdown also suppresses caspase-3 enzymatic activity and reduces TUNEL-positive cell death, indicating that circFOXO3 promotes neuronal loss through the cytochrome c/caspase-3 mitochondrial (intrinsic) apoptosis pathway [93].

While circRNAs have traditionally been characterized primarily as non-coding sponges for miRNAs, emerging evidence suggests that certain circRNAs are translated into functional peptides or proteins [94]. Translation is typically initiated by internal ribosome entry sites (IRES) or short IRES-like elements and by N6-methyladenosine (m6A) modifications, which recruit initiation factors independently of a 5′ cap [94,96] (Figure 3). CircRNAs whose open reading frame lacks an in-frame stop codon can additionally undergo rolling-circle translation, producing repetitive protein products and adding to proteome diversity—though most circRNA-derived peptides are of low abundance [94,96]. Because cap-independent translation is favored under cellular stress, circRNA-derived proteins tend to be produced under conditions such as hypoxia [94,96]. Importantly, hypoxia is not merely a generic cellular stress but a pathophysiological condition of direct relevance to AD [97]. Chronic hypoxia biases APP processing toward the amyloidogenic route: it lowers both the abundance and the maturation of the α-secretase ADAM10 and reduces expression of the related sheddase ADAM17 (17), thereby reducing non-amyloidogenic cleavage of APP and favoring Aβ generation [98]. This hypoxia-induced suppression of ADAM10 converges on the same α-secretase pathway regulated by circHDAC9 [29], suggesting that hypoxia and circRNA dysregulation may cooperatively shift APP processing toward amyloidogenesis. Future research will hopefully unravel such molecular mechanistic insights.

**Figure 4:**
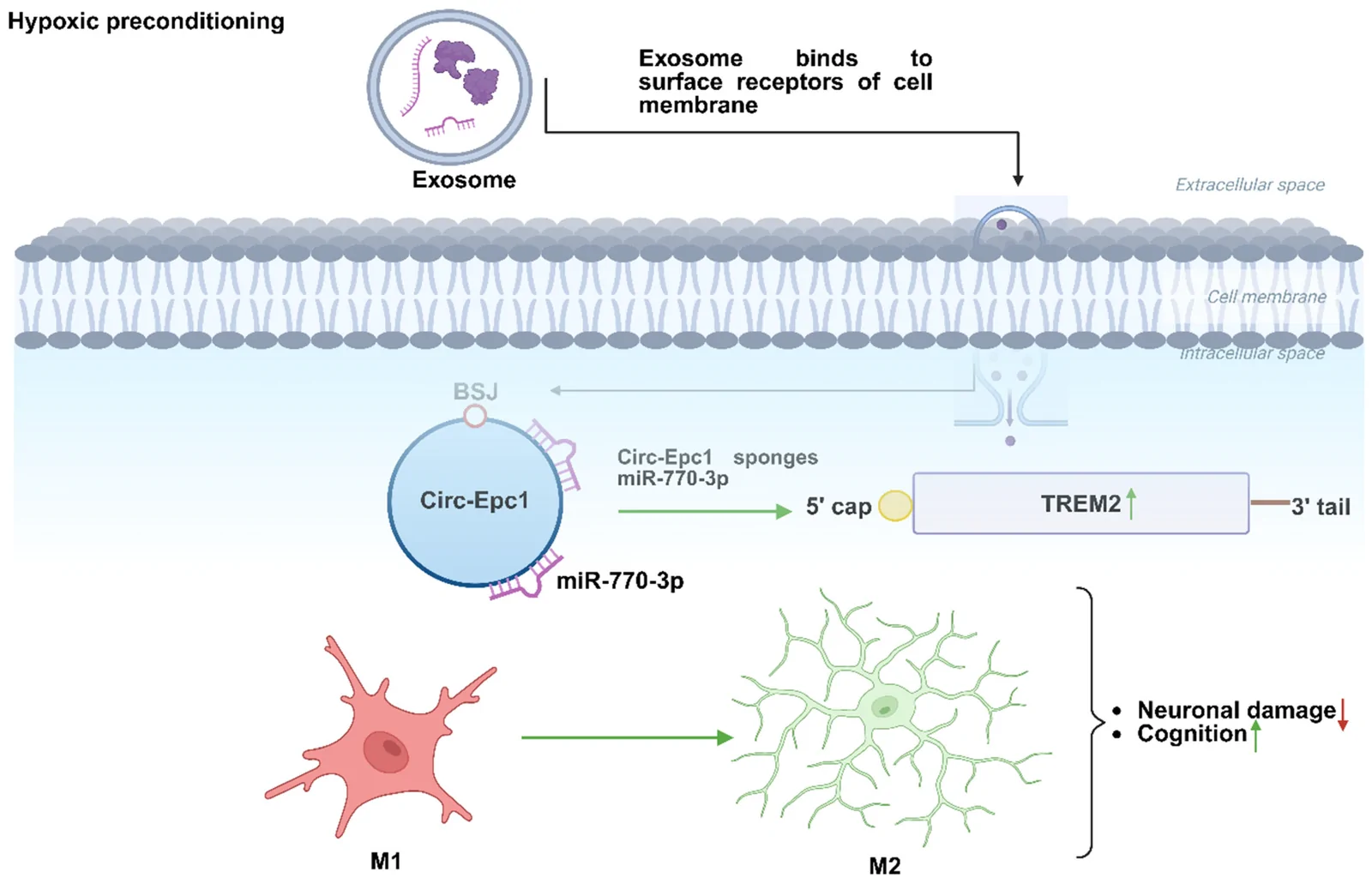
Hypoxic preconditioning of adipose-derived stem cells enriches circ-Epc1 in exosomes; delivered to APP/PS1 mice, circ-Epc1 sponges miR-770-3p to de-repress TREM2, shifting microglia M1 → M2 and improving cognition (Created in BioRender. Yuksel, M. (2026) https://BioRender.com/v3uon67). Extracellular vesicles, specifically exosomes, are released following a hypoxic preconditioning stimulus and serve as critical delivery vehicles for the circRNA, Circ-Epc1 [99]. These exosomes traverse the extracellular space and dock onto complementary surface receptors on the target recipient cell membrane [99]. Following receptor binding and subsequent endosomal internalization or membrane fusion, the exosomal cargo is released into the intracellular space [99]. Within the cytosol, Circ-Epc1—structurally defined by its covalently closed continuous loop and characteristic BSJ—exerts post-transcriptional regulatory control by functioning as a competing endogenous RNA (ceRNA) [99]. Circ-Epc1 physically sponges miR-770-3p via complementary binding sites, thereby sequestering the miRNA and preventing it from interacting with its downstream targets [99]. This ceRNA network effectively relieves the miR-770-3p-mediated translational repression of the TREM2 mRNA [99]. The resulting de-repression leads to a significant upregulation of TREM2 expression [99]. At the cellular level, amplified TREM2 signaling drives the phenotypic polarization of microglia/macrophages away from the pro-inflammatory, neurotoxic M1 state and toward the anti-inflammatory, tissue-remodeling M2 state [99]. Ultimately, this molecular cascade and subsequent immunomodulation converge to attenuate hypoxia-induced neuronal damage and promote the recovery of cognitive function [99].

Hypoxia also intersects circRNA biology in a manner that carries therapeutic promise. Hypoxic preconditioning of adipose-derived stem cells enriches the circRNA circ-Epc1 (mmu_circ_0000842) within their secreted exosomes; when these exosomes are delivered to APP/PS1 mice, circ-Epc1 sponges miR-770-3p to de-repress TREM2 (Triggering receptor expressed on myeloid cells 2), shifting hippocampal microglia from the pro-inflammatory M1 toward the anti-inflammatory M2 state, attenuating neuronal damage and improving cognition [99] (Figure 4). This reflects an exogenous, exosome-delivered intervention rather than endogenous hypoxia-driven circRNA production within the AD brain; nonetheless, it suggests that hypoxia-responsive circRNAs can be harnessed to recalibrate neuroinflammatory tone and cognitive outcomes in AD models.

## 4. CircRNAs as Diagnostic Biomarkers

Several neurodegenerative and non-neurodegenerative disorders beyond AD can cause dementia—including dementia with Lewy bodies, vascular dementia, and Parkinson’s disease—so that accurate discrimination between dementia subtypes remains a major diagnostic challenge in routine practice [18]. Current diagnostic algorithms still rely heavily on cognitive testing and structural imaging, by which point substantial neurodegeneration has typically already occurred [5]. This limitation has only grown more consequential with the recent approval of disease-modifying anti-amyloid monoclonal antibodies such as lecanemab and donanemab, whose clinical benefit is modest and largely confined to early, biomarker-confirmed disease [5,6]. Therefore, the emergence of these therapies further underscores the need for earlier and more accurate diagnosis [5,6]. In the pivotal CLARITY AD trial, lecanemab slowed decline on the Clinical Dementia Rating–Sum of Boxes (CDR-SB) by 27% over 18 months in participants with mild cognitive impairment or mild AD dementia [100]. Similarly, donanemab reduced decline in the TRAILBLAZER-ALZ 2 trial, with the greatest benefit observed in participants with lower baseline tau burden and earlier-stage disease [101]. Although these findings indicate that amyloid-targeting therapies can modify the progression of clinical and cognitive impairment, the magnitude of benefit remains modest and appears to diminish with more advanced pathology [100,101]. Their use is also constrained by amyloid-related imaging abnormalities (ARIA), including vasogenic edema (ARIA-E) and microhemorrhage or superficial siderosis (ARIA-H), which require baseline and serial MRI monitoring [100,101]. ARIA risk is substantially higher in APOE ε4 homozygotes, making genotype and safety considerations particularly relevant to treatment selection [100,101]. Overall, these findings indicate that current disease-modifying therapies provide measurable but limited benefit within a relatively narrow therapeutic window and do not halt the underlying neurodegenerative process [100,101]. These limitations highlight the need for sensitive molecular biomarkers capable of detecting AD in earlier stages.

Current diagnostic efforts encompass complementary modalities, including CSF biomarkers (Aβ42/40 ratio, p-tau181, p-tau217), amyloid- and tau-positron emission tomography (PET) imaging, retinal biomarkers, and digital cognitive assessments [102,103,104,105,106,107]. A notable recent shift, however, has been the emergence of non-invasive, blood-based biomarkers that avoid the procedural burden of lumbar puncture and the cost and accessibility limitations of PET imaging—exemplified by plasma p-tau217, which distinguishes pathologically confirmed AD from non-AD with an AUC of 0.88–0.90 and may rise up to 20 years before mild cognitive impairment onset in inherited AD [37]. Similarly, in the AB255 study (83 cognitively normal individuals and 145 amnestic mild cognitive impairment [MCI] patients), a low plasma Aβ42/40 ratio predicted a 70% increased risk of progression to dementia over two years [102].

In this context, circRNAs have emerged as promising blood-based molecular biomarkers, with several properties that may support their use in place of or alongside conventional RNA- and protein-based markers, as discussed in the “CircRNA Biogenesis section”. Moreover, unlike p-tau or Aβ species, which primarily reflect individual pathological processes, circRNAs may provide information on multiple AD-related molecular processes through distinct regulatory mechanisms. For instance, hsa_circRNA_003022 originates from PITRM1 (pitrilysin metallopeptidase 1), a mitochondrial metallopeptidase that physiologically degrades the mitochondrial fraction of Aβ and whose dysfunction has been linked to mitochondrial Aβ accumulation and amyloidotic neurodegeneration [36,108] (Table 1). Collectively, these findings suggest that some circRNA biomarkers may have biological links to AD-related pathways beyond their statistical association with disease status, supporting their potential diagnostic relevance. A summary of the diagnostic circRNA candidates is provided in Table 1.

**Table 1:** Summary of candidate circRNA biomarkers for Alzheimer’s Disease, detailing specimen sources, expression patterns, and diagnostic metrics.

CircRNA Name	Sample Type	Expression Pattern	Patient Cohort/Cell Line	Diagnostic Value (AUC)	Target Genes/Pathways	Ref.
**hsa_circRNA_050263**	Human Plasma	Up-regulation (significant increase in both males and females)	Patient cohort: 26 patients with AD, 29 healthy persons, 5 patients with MCI	0.980	ATP13A1	[36]
**hsa_circRNA_102619**	Human Plasma	Down-regulation (significant decrease in just female)	Patient Cohort: 26 patients with AD, 29 healthy people, 5 patients with MCI	0.817	NOL10	[36]
**hsa_circRNA_102049**	Human Plasma	Down-regulation (significant decrease in both males and females)	Patient Cohort: 26 patients with AD, 29 healthy persons, 5 patients with MCI	0.829	TADA2A	[36]
**hsa_circRNA_100837**	Human Plasma	Up-regulation (significant increase in both males and females)	Patient Cohort: 26 patients with AD, 29 healthy persons, 5 patients with MCI	0.855	-	[36]
**hsa_circRNA_003022**	Human Plasma	Up-regulation (significant increase in both males and females)	Patient Cohort: 26 patients with AD, 29 healthy people, 5 patients with MCI	0.710	PITRM1	[36]
**hsa_circRNA_403959**	Human Plasma	Up-regulation (significant increase in both males and females)	Patient Cohort: 26 patients with AD, 29 healthy people, 5 patients with MCI	0.714	BRAF	[36]
**circ_0000497**	CSF and Bioinformatics	Up-regulation	Bioinformatics: GSE104704 (12 patients with AD, 10 healthy people) Patient Cohort: Clinical CSF samples from both AD patients and healthy controls	0.758	-	[104]
**circ_0001519**	CSF and Bioinformatics	Up-regulation	Bioinformatics: GSE104704 (12 patients with AD, 10 healthy people) Patient Cohort: Clinical CSF samples from both AD patients and healthy controls	0.775	-	[104]
**circ_0001535**	In Vitro, CSF and Bioinformatics	Up-regulation	In Vitro: SH-SY5Y Bioinformatics: GSE104704 (12 patients with AD, 10 healthy people) Patient Cohort: Clinical CSF samples from both AD patients and healthy controls	0.900	circ_0001535↑/E2F1↑/DHFR↑ (promoting apoptosis)	[104]
**circ_0002454**	CSF and Bioinformatics	Up-regulation	Bioinformatics: GSE104704 (12 patients with AD, 10 healthy people) Patient Cohort: Clinical CSF samples from both AD patients and healthy controls	0.733	-	[109]
**circ_0001380**	CSF and Bioinformatics	Up-regulation	Bioinformatics: GSE104704 (12 patients with AD, 10 healthy people) Patient Cohort: Clinical CSF samples from both AD patients and healthy controls	0.867	-	[109]
**circ_0087960**	CSF and Bioinformatics	Up-regulation	Bioinformatics: GSE104704 (12 patients with AD, 10 healthy people) Patient Cohort: Clinical CSF samples from both AD patients and healthy controls	0.758	-	[109]
**circ_0006916**	CSF and Bioinformatics	Down-regulation (female-specific)	Bioinformatics: GSE104704 (12 patients with AD, 10 healthy people) Patient Cohort: Clinical CSF samples from both AD patients and healthy controls	0.875	-	[109]
**circ_0131934**	Bioinformatics	Down-regulation	Bioinformatics: GSE104704 (12 patients with AD, 10 healthy people) Patient Cohort: Clinical CSF samples from both AD patients and healthy controls	0.858	-	[109]
**circ_0099634**	Bioinformatics	Down-regulation	Bioinformatics: GSE104704 (12 patients with AD, 10 healthy people) Patient Cohort: Clinical CSF samples from both AD patients and healthy controls	0.733	-	[109]
**circ_0017248**	CSF	Down-regulation	SH-SY5Y, Gene Expression Omnibus (GEO) Database Patient Cohort: Clinical CSF samples from both AD patients and healthy controls	0.800	-	[109]
**circ_0006837**	CSF	Down-regulation	SH-SY5Y, Gene Expression Omnibus (GEO) Database Patient Cohort: Clinical CSF samples from both AD patients and healthy controls	0.800	-	[109]
**hsa_circ_0003391 (The expression of hsa_circ_0003391 had a specific decrease in the peripheral blood of patients with AD compared to those with other types of dementia)**	Human Plasma and Whole blood cells	Down-regulation	50 patients with AD, 20 patients with dementia with Lewy Bodies, 40 patients with vascular dementia, 50 healthy individuals as control group	Plasma: 0.7283 Whole blood cells: 0.7089	hsa_circ_0003391↓/miR-574-5p↑ (increased APP production)	[110]

NOL10: nuclear protein 10; TADA2A: transcriptional adaptor 2A; BRAF: B-raf proto-oncogene, serine/threonine kinase; E2F1: E2 transcription factor 1; DHFR: dihydrofolate reductase; GSE/GEO: gene expression omnibus.

## 5. CircRNAs in AD Pathogenesis

### 5.1. CircRNAs and Amyloid-β Plaque Formation

**Figure 5:**
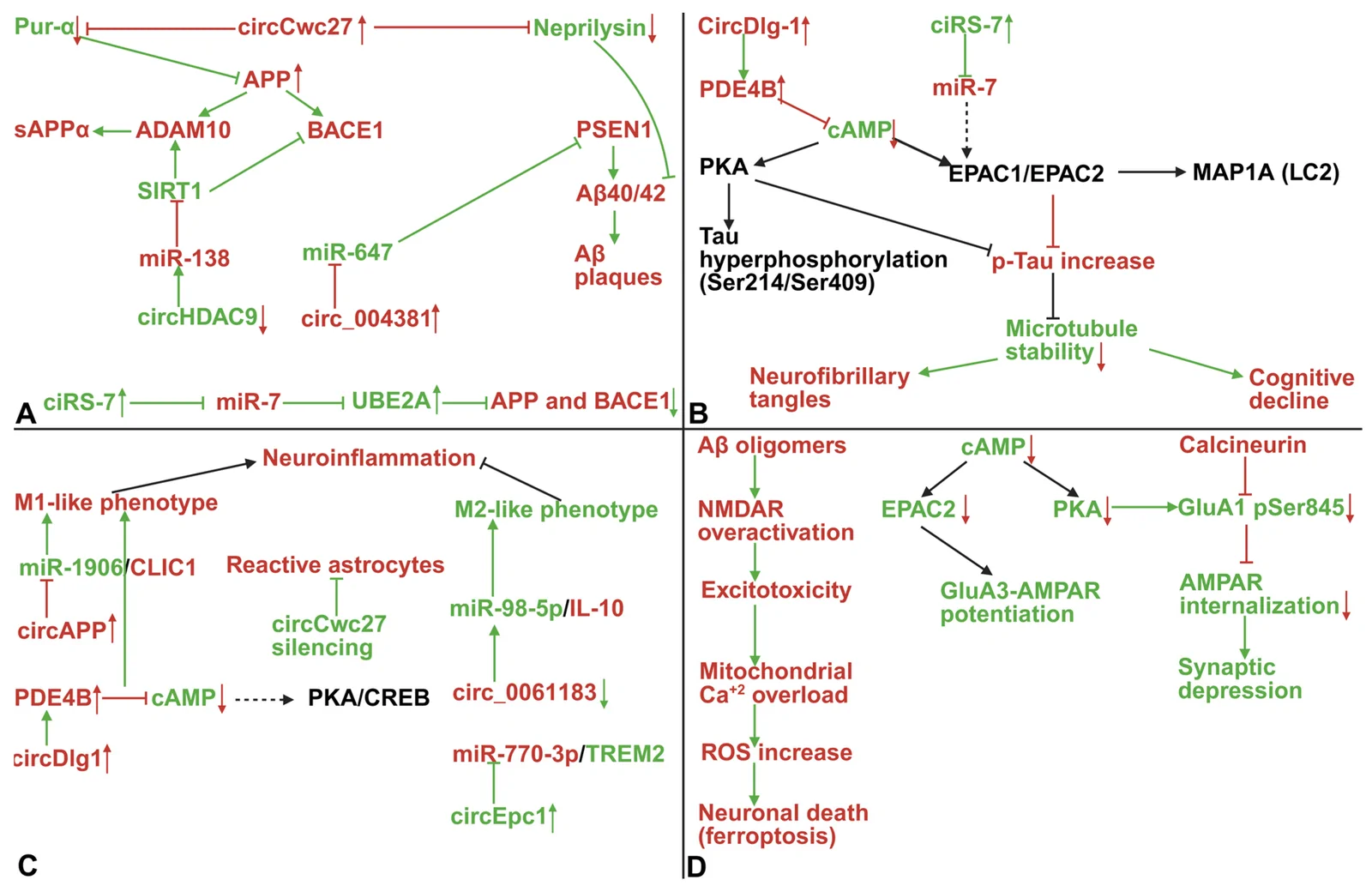
CircRNA-mediated regulation of major AD-related pathways (Created in BioRender. Yuksel, M. (2026) https://BioRender.com/bjy0953). (**A**) Aβ production and clearance. circCwc27↑ inhibits Pur-α and reduces neprilysin, thereby increasing APP availability while decreasing Aβ degradation [27]. circHDAC9↓ increases miR-138 activity, reduces SIRT1, and shifts APP processing away from ADAM10-mediated non-amyloidogenic processing [29]. circ_0004381↑ suppresses miR-647, increasing PSEN1 and Aβ40/42 production [30]. ciRS-7 regulates the miR-7/UBE2A pathway, linking reduced proteostatic clearance of APP/BACE1 with increased Aβ burden [25]. (**B**) Tau-related signalling. circDlg1↑ increases PDE4B and reduces cAMP, potentially altering PKA- and EPAC-dependent signalling. PKA contributes to tau phosphorylation, whereas EPAC1/2 interact with MAP1A-LC2 and may influence microtubule regulation [35]. ciRS-7/miR-7 represents a potential upstream regulator of EPAC signalling. Increased p-tau and reduced microtubule stability contribute to neurofibrillary tangle formation and cognitive decline [111]. (**C**) Neuroinflammation. circAPP↑ promotes M1-like microglial responses through the miR-1906/CLIC1 pathway [33], while circDlg1↑ reduces cAMP through PDE4B and is associated with pro-inflammatory signalling [35]. In contrast, circ_0061183 and exosomal circEpc1 support M2-like responses through miR-98-5p/IL-10 [30] and miR-770-3p/TREM2-related pathways, respectively [99]. circCwc27 silencing is also associated with reduced astrocytic activation [27]. (**D**) Synaptic dysfunction and ferroptotic susceptibility. Aβ oligomers promote NMDAR overactivation, excitotoxicity, mitochondrial Ca^2+^ overload, ROS generation, and neuronal death [127]. Reduced cAMP/EPAC2 signalling impairs GluA3-AMPAR potentiation, while reduced PKA activity and increased calcineurin-dependent dephosphorylation of GluA1 at Ser845 promote AMPAR internalisation and synaptic depression [127]. Arrows indicate activation or promotion, inhibitory bars indicate suppression, and dashed lines indicate proposed or indirect relationships. ↑ and ↓ indicate increased and decreased expression or activity, respectively.

Aβ levels in the brain are determined by the balance between Aβ production through amyloidogenic APP processing and its removal through enzymatic degradation and cellular clearance [111]. APP processing itself can proceed through either the non-amyloidogenic or amyloidogenic pathway [112]. In the non-amyloidogenic pathway, α-secretase cleaves APP within the Aβ sequence itself, restricting Aβ production and releasing the neurotrophic soluble fragment sAPPα [113] (Figure 5). In the amyloidogenic pathway, APP is sequentially cleaved by β- and γ-secretases at sites flanking the Aβ region, generating pathogenic Aβ peptides [114] (Figure 5). The balance between these competing pathways determines net Aβ production, and its disruption is a central event in AD pathogenesis [114]. Although the physiological functions of APP and Aβ mechanisms are not fully elucidated, accumulating evidence suggests that they play important roles in neuronal development, survival, synaptic plasticity, and signal transduction [111,114]. The fundamental pathological problem caused by Aβ lies in its intrinsic conformational instability [114]. Under physiological conditions, monomeric Aβ adopts a predominantly disordered random-coil conformation [115]. The aggregation-prone Aβ42 isoform—which differs from the more soluble Aβ40 by only two additional C-terminal hydrophobic residues (Ile41–Ala42)—readily transitions into β-sheet–rich conformations, nucleating a stepwise self-assembly cascade that proceeds from soluble oligomers through protofibrils to insoluble fibrillar plaques [115,116]. These soluble oligomeric intermediates, rather than mature plaques, are now regarded as the principal neurotoxic species, since plaque burden correlates poorly with cognitive decline whereas soluble oligomer levels track more closely with disease severity [116,117]. CircRNAs may influence both processes through distinct mechanisms (Figure 5).

CircRNAs can influence Aβ production through ceRNA-mediated regulation of amyloidogenic APP processing. For example, circ_0004381 sponges miR-647 to elevate PSEN1, the catalytic subunit of γ-secretase that executes the second cleavage step generating mature Aβ peptides [30] (Figure 5). circHDAC9 may reduce amyloidogenic APP processing by sponging miR-138 and increasing SIRT1 expression. This promotes ADAM10-mediated non-amyloidogenic processing and thereby limits Aβ production [29] (Figure 5). Reduced circHDAC9 in AD and MCI may increase miR-138 activity, decrease SIRT1 expression, and enhance amyloidogenic APP processing, resulting in increased Aβ production [29] (Figure 5). Other circRNAs can act downstream of Aβ rather than directly regulating APP processing. For example, circLPAR1 is upregulated in AD serum and Aβ-exposed neuronal cells, where it regulates the miR-212-3p/ZNF217 (Zinc finger protein 217) axis. circLPAR1 silencing improves cell viability and reduces apoptosis, inflammatory responses and ROS production [118]. Additional ceRNA pathways associated with Aβ pathology are summarised in Table 2.

Beyond ceRNA activity, circRNAs can disrupt Aβ homeostasis through direct protein interactions. CircCwc27, upregulated in both AD mouse models and human postmortem brain, physically sequesters Pur-α—a purine-rich DNA/RNA-binding transcription factor that normally represses APP expression and promotes synaptic integrity—away from its nuclear targets [27]. The resulting derepression simultaneously raises APP transcription and downregulates neprilysin, the principal extracellular endopeptidase responsible for enzymatic Aβ degradation [27]. Together, these changes may increase Aβ production while reducing its clearance. Accordingly, circCwc27 knockdown can reduce plaque burden, preserve synaptic proteins, and rescue cognitive performance, identifying circCwc27 as a hub coordinating two opposing arms of Aβ homeostasis [27] (Figure 5).

The clearance of Aβ homeostasis is comparatively less studied but is represented by several transcripts. ciRS-7 acts on this arm in two ways: as a miR-7 sponge, it preserves expression of the ubiquitin-conjugating enzyme UBE2A required for proteasomal Aβ clearance [25], and it additionally lowers APP and BACE1 protein levels by promoting their degradation through the proteasome and lysosome in an NF-κB-dependent manner without altering their transcription [26] (Figure 5). The human APP-derived circRNA hsa_circ_0007556 is also reduced in the entorhinal cortex of patients with AD, where its expression is inversely associated with Aβ burden [119]. Notably, this reduction occurs without a corresponding change in linear APP mRNA, indicating differential regulation of the circular and linear APP transcripts [119].

### 5.2. CircRNAs and Tau Pathology in Alzheimer’s Disease

Tau aggregation is a major pathological feature of AD and other neurodegenerative disorders [120]. Tau is a microtubule-associated protein (MAP) whose function is regulated by reversible phosphorylation, including at PKA-sensitive sites such as Ser214 and Ser409 [121]. In AD, increased kinase activity and reduced phosphatase activity promote tau hyperphosphorylation, impairing microtubule binding and contributing to cytoskeletal instability and neurofibrillary tangle formation [121] (Figure 5). cAMP signalling may influence this process through both PKA- and EPAC-dependent pathways. EPAC1 and EPAC2 interact with light chain 2 of MAP1A (1A) [122], while altered cAMP/EPAC signalling has been reported in AD [123]. Pharmacological elevation of cAMP with the PDE4 inhibitor rolipram reduces tau phosphorylation and amyloid burden in 3 × Tg-AD mice through pathways involving PKA/26S proteasome and EPAC/ERK (Extracellular signal-regulated kinase) signalling [124]. These findings are relevant to circDlg1, which regulates the PDE4B/cAMP/PKA/CREB pathway [35] (Table 2), although its direct effect on tau has not been examined.

Another potential circRNA-related pathway involves ciRS-7. ciRS-7 regulates miR-7 [95], which can repress EPAC1 in non-neuronal cells [125], suggesting a possible ciRS-7/miR-7/EPAC1 link to microtubule-associated signalling (Figure 5). However, this pathway has not been demonstrated in AD neurons, and its effect on tau remains unknown. More broadly, direct regulation of tau by AD-associated circRNAs has not yet been established. Current evidence instead supports indirect connections through pathways involved in tau phosphorylation, proteostasis, microtubule regulation, and cAMP signalling, which are discussed further in the Discussion and Outlook section.

### 5.3. CircRNAs and Neuroinflammation

Chronic neuroinflammation is closely associated with Aβ and tau pathology in AD and involves sustained changes in microglial and astrocytic function [11]. Microglia can adopt diverse functional states that are commonly described using M1-like and M2-like phenotypes. M1-like states are associated with increased production of pro-inflammatory cytokines and ROS, whereas M2-like states are associated with anti-inflammatory responses and the clearance of cellular debris and Aβ [12]. However, the M1/M2 classification does not fully represent the heterogeneity of microglial states in AD [13]. Functional outcomes, including changes in Aβ phagocytosis and cognition, should therefore be considered alongside changes in M1- and M2-associated markers. CircRNAs have been identified as upstream regulators of this balance in both directions.

Several AD-associated circRNAs have been implicated in the regulation of microglial inflammatory responses. circ_0061183 is reduced in the brain of AD mice and in the blood of individuals with cognitive impairment, and its loss promotes microglial activation and a shift toward an M1-like phenotype while reducing M2-like-associated responses [34]. Mechanistically, circ_0061183 regulates miR-98-5p and downstream IL-10 (Interleukin 10) and TGF-β (Transforming growth factor β) signalling, providing a potential pathway through which its reduction may contribute to neuroinflammation (Figure 5) [34]. In contrast, the murine APP-derived circAPP is increased in the hippocampus of APP/PS1 mice and promotes pro-inflammatory microglial responses through the miR-1906/CLIC1 pathway [33]. CLIC1 can translocate to the plasma membrane following Aβ exposure and contribute to microglial inflammatory activity, whereas circAPP silencing reduces M1-like markers, increases M2-like-associated responses, and enhances microglial Aβ phagocytosis [33] (Figure 5).

Microglial responses can also be modulated by exogenously delivered circRNAs. Exosomes derived from hypoxia-preconditioned adipose-derived stem cells are enriched in circ-Epc1, and their administration to APP/PS1 mice promoted an anti-inflammatory microglial phenotype through the miR-770-3p/TREM2 pathway, accompanied by reduced neuronal damage and improved cognitive function [97]. CircDlg1 can provide a distinct mechanism involving cAMP signalling, as explained previously. In microglia, circDlg1 regulates the PDE4B/cAMP/PKA/CREB pathway, while increased circDlg1 expression is associated with enhanced neuroinflammation and cognitive impairment [35].

The role of circRNAs in astrocytic responses is less well characterised than their role in microglia. Astrocytes contribute to Aβ clearance, brain iron homeostasis and neuronal antioxidant support, while reactive astrocytic states can also be associated with inflammatory and neurotoxic activity [14,126]. In one study, circCwc27 silencing reduced both microglial and astrocytic activation [27]; however, astrocytes were not examined independently, so this finding does not establish a direct or cell-autonomous effect of circCwc27 in astrocytes. More broadly, direct evidence for astrocyte-specific regulation by AD-associated circRNAs remains limited, although several circRNAs implicated in neuroinflammatory processes are summarised in Table 2.

### 5.4. CircRNAs and Synaptic Dysfunction

A central downstream consequence of this synaptic engagement is dysregulation of the NMDA receptor—a glutamate-gated ionotropic receptor essential for Ca^2+^ (calcium)-dependent synaptic plasticity, long-term potentiation (LTP), and memory [127]. Aβ oligomers chronically over-activate extrasynaptic NMDA receptors, causing pathological Ca^2+^ influx (excitotoxicity) that drives mitochondrial Ca^2+^ overload, opening of the mitochondrial permeability transition pore, collapse of the membrane potential, and overproduction of ROS, eventually resulting in neuronal death, particularly through ferroptosis [127] (Figure 5). This same Ca^2+^ dysregulation reshapes AMPA-receptor signaling: by activating the Ca^2+^-dependent phosphatase calcineurin, it dephosphorylates the GluA1 subunit at Ser845—the residue whose phosphorylation by PKA stabilizes AMPA receptors at the synaptic surface—promoting receptor internalization, synaptic depression, and dendritic spine loss [127]. These events converge on cAMP, a key second messenger for synaptic plasticity and memory that signals through PKA and the exchange protein EPAC2 [127] (Figure 5). Beyond the PKA–GluA1 axis, cAMP supports a distinct, NMDA-independent form of postsynaptic potentiation mediated by EPAC2 and GluA3-containing AMPA receptors—a pathway that Aβ selectively impairs [123]. Consistent with a permissive role for cAMP, cAMP levels and EPAC2 expression are reduced in the AD hippocampus and cortex and correlate with Aβ burden, whereas pharmacologically raising cAMP restores synaptic plasticity and memory in AD models [123]. Collectively, these interconnected pathological processes—impaired protein homeostasis, NMDA receptor-mediated excitotoxicity, AMPA-receptor loss, disrupted cAMP/EPAC2 signaling, oxidative stress, and mitochondrial impairment—progressively impair synaptic integrity and trigger neuronal death, producing the synaptic loss, hippocampal and cortical atrophy, and progressive cognitive decline that clinically define AD [118,121] (Figure 5).

Silencing circCwc27 preserves the synaptic proteins MAP2 (microtubule associated protein 2), PSD95 (post-synaptic density protein 95), synapsin-1 and synaptophysin, together with reducing Aβ plaque burden [27]. Reduced circHDAC9 is associated with decreased dendritic spine density and impaired spatial learning and memory, in addition to its effects on APP processing [29]. Similarly, downregulation of circ-Bptf contributes to synaptic dysfunction and cognitive impairment through the miR-138-5p/p62 pathway [32]. RNA modification provides an additional level of circRNA regulation relevant to synaptic dysfunction. Increased circRIMS2 expression in four-month-old APP/PS1 mice is dependent on METTL3 (Methyltransferase-like 3)-mediated m6A modification [31]. m6A modification can also promote cap-independent translation of circRNAs [98], indicating that m6A can regulate circRNA function through mechanisms affecting both RNA abundance and translation. However, m6A-dependent translation of circRIMS2 has not been demonstrated.

### 5.5. Other Pathological Mechanisms

Beyond the mechanisms discussed above, circRNAs are associated with several processes that may influence multiple aspects of AD pathology. These include oxidative stress and regulated cell death, protein homeostasis and endolysosomal dysfunction with associated mitochondrial alterations, RNA editing and circRNA translation, and sex- and brain region-specific patterns of circRNA dysregulation (Table 2).

Oxidative stress and regulated cell death are frequently reported following circRNA manipulation in AD models, although the specific cell-death mechanisms are often not established. circFoxO3 provides a more clearly characterised example involving apoptosis [93]. In hippocampal HT22 cells, glutamate exposure increases ROS production and circFoxO3 expression, while circFoxO3 interacts with the pro-apoptotic protein BimEL [93]. These changes are accompanied by mitochondrial membrane depolarisation, cytochrome c release and caspase-3 activation and are reduced by circFoxO3 silencing [93]. Other circRNAs have been associated with broader oxidative and cell-death phenotypes. circ_0004381 overexpression increases oxidative stress, mitochondrial dysfunction, apoptosis and pro-inflammatory microglial responses [30], whereas silencing of circLPAR1 [118], hsa_circ_0049472 [128] and circ_0003611 [129,130] reduces oxidative stress and cell-death-related changes in their respective experimental models (Table 2). These findings require careful interpretation because apoptosis, pyroptosis and ferroptosis are mechanistically distinct forms of regulated cell death, while commonly measured endpoints such as cell viability, total ROS and inflammatory cytokines are not sufficient to distinguish among them.

Protein homeostasis and endolysosomal dysfunction provide another potential connection among several circRNA-regulated processes in AD (Table 2). The clearance of abnormal and aggregation-prone proteins involves the ubiquitin–proteasome system, autophagy–lysosomal pathways and endoplasmic reticulum quality control, all of which are disrupted in AD [131]. Impaired lysosomal acidification has also been implicated in neurodegenerative disease [132]. Several AD-associated circRNAs regulate components of these pathways. ciRS-7 influences APP and BACE1 degradation through proteasomal and lysosomal mechanisms [25,26], while circ-Bptf regulates p62, a selective autophagy receptor involved in the recognition and clearance of ubiquitinated proteins [32]. circRIMS2 increases UBE2K expression and promotes UBE2K-dependent ubiquitination and degradation of GluN2B [31]; UBE2K has also been associated with impaired proteasomal activity, although this broader effect has not been demonstrated downstream of circRIMS2. The endolysosomal system is also connected to mitochondrial regulation. Lysosomal mTORC1 (Mechanistic target of rapamycin complex 1) participates in nutrient-dependent regulation of mitochondrial activity and mitochondrial DNA synthesis, whereas Aβ oligomers disrupt this signalling through a tau-dependent mechanism [133,134]. CircRNA regulation of this lysosome–mitochondria pathway has not been investigated.

RNA editing and circRNA translation represent additional mechanisms through which circRNAs may contribute to AD pathology. circMAN2A1 provides a notable example: ADAR1/ADAR2-mediated A-to-I editing can generate start codons that are out of frame with the linear MAN2A1 transcript, enabling translation of chimeric peptides that are absent from the canonical MAN2A1 protein and increase with AD severity [28]. This finding indicates that circRNAs may contribute to AD not only by regulating gene expression but also through the production of circRNA-derived peptides.

Sex and brain region may influence patterns of circRNA dysregulation in AD. HOMER1-derived circRNAs are downregulated in the entorhinal cortex of female patients with AD, although their inverse association with cortical Aβ burden is observed in both sexes [4]. This finding is relevant to broader evidence for sex differences in AD and raises the possibility that sex-dependent biological factors contribute to circRNA regulation. Oestrogen signalling is one potential factor because it has been associated with cognitive function and AD risk [135,136] and can regulate processes including autophagy and ferroptosis [137]. However, a relationship between oestrogen signalling and circHOMER1 or other sex-associated circRNAs in AD has not been established.

**Table 2:** Summary of circRNAs involved in AD pathogenesis, detailing their regulatory mechanisms, specific miRNA targets, and downstream signaling pathways.

CircRNA Name	Sample Type	Expression Pattern	Patient Cohort/Cell Line	Target Genes/Pathways	Functional Outcome	Ref.
**circLPAR1**	In Vitro and Human Serum Samples	Up-regulation	In Vitro: CHP-212, IMR-32 Patient Cohort: 37 AD patients, 37 healthy controls	circLPAR1↑/miR-212-3p↓/ZNF217 ↑/Aβ↑	Drives Aβ-induced neuronal neurotoxicity. Silencing circLPAR1 reverses AD-like injury by restoring viability, suppressing apoptosis, reducing inflammation, and decreasing ROS	[118]
**circRIMS2**	In vitro and In vivo	Up-regulation	In vitro: Mouse primary cortical neurons, N2a, HEK293 In vivo: APP/PS1 mice, 3x Tg mice (triple transgenic), WT C57BL/6 mice (wild type cage 57 black 6)	circRIMS2↑/miR-3968↓/UBE2K ↑/GluN2B (a NMDA receptor) ↓	Decreased dendritic spine density Significant learning and memory deficits	[31]
**circCwc27**	In vivo, In vitro and Ex vivo	Up-regulation	In Vitro: SH-SY5Y, HEK293T, Primary mouse cortical neurons, astrocytes, and microglia In Vivo: APP/PS1 mice, Wild type (WT) C57BL/6J mice Patient cohort: 6 plasma samples of patients with AD, 6 plasma samples of healthy controls, 13 postmortem human brain tissue samples diagnosed with AD, 8 postmortem human brain tissue samples not diagnosed with dementia	circCwc27↑/Pur-α↓/Aβ↑	Silencing circCwc27 reverses AD-like injury by reducing Aβ plaques, preserving synapse-associated proteins (MAP2, PSD95, synapsin-1, and synaptophysin), attenuating neuroinflammation (markedly reduced activation of microglia and astrocytes), and improving cognition	[27]
**circHOMER1**	Ex Vivo	Down-regulation	Patient Cohort: 28 postmortem human brain tissue samples diagnosed with AD, and 16 postmortem human brain tissue not diagnosed with dementia (23 females and 21 males)	circHOMER1↓/HOMER1 ↓/Aβ ↑	The downregulation of circHOMER1 is closely associated with the severity of AD neuropathological changes in females.	[4]
**circAPP**	In Vitro and In Vivo	Up-regulation	In Vitro: Primary neuron and glial cultures (derived from 1-day-old C57BL/6J mice), BV-2, C8-D1A, N2a In Vivo: APP/PS1 mice, WT C57BL/6J mice	circAPP↑/miR-1906↓/CLIC1↑/Aβ↑	Silencing circAPP reverses AD pathology by shifting microglia from M1-phenotype to M2-phenotype, enhancing microglial phagocytosis, improving spatial memory Microglia specific	[33]
**circ_0005835**	In Vitro and Human venous blood	Up-regulation	In Vitro: BV-2, SH-SY5Y, and Primary neural stem cells (Isolated from the telencephalon of E14.5 rats) Patient Cohort: 30 plasma samples of patients with AD, 30 plasma samples of healthy control	circ_0005835↑/miR-576-3p↓	Silencing circ_0005835 mitigates AD-like cellular injury by reducing neuroinflammation, promoting neural stem cell proliferation, driving neuronal differentiation	[138]
**circMAN2A1**	In Vitro and Ex Vivo	Up-regulation	In Vitro: HEK293 Patient Cohort: 7 human postmortem tissue samples derived from the entorhinal and temporal cortex	circMAN2A1↑/ADAR1 and ADAR2↑/N-terminal catalytic domain of linear MAN2A1 and predicted Rho binding domain	Progressive AD-stage correlation Pathological protein production	[28]
**circHDAC9**	In vitro, in vivo, human serum samples	Down-regulation	In Vitro: N2a In Vivo: APP/PS1 mice Patient Cohort: Serum samples of 7 AD patients, 10 amnestic MCI patients, 17 serum samples	circHDAC9↓/miR-138↑/Sirt1↓/ADAM10↓/Aβ↑	Abnormal APP processing Reduction in dendritic spine density Severe deficits in spatial learning and memory	[29]
**circ_0061183**	In Vitro, In Vivo, Human PBMC and Plasma Samples	Down-regulation	In Vitro: HMC3, HEK293 In Vivo: WT C57BL/6J mice, 5xFAD mice (Familial AD) Patient Cohort: 100 people over 65 years of age	circ_0061183↓/miR-98-5p↑/Aβ↓	Silencing of circ_0061183 exacerbates microglial activation	[34]
**hsa_circ_0131235**	Ex vivo	Up-regulation	Patient Cohort: 100 postmortem human brain tissue diagnosed with AD	-	It is not correlated with cognitive capability/decline indicating its functional role is tied specifically to the physiological accumulation of AD neuropathology rather than the resulting symptomatic dementia.	[139]
**circ_0002945**	In Vitro and Human serum samples	Up-regulation	In Vitro: SK-N-SH, Human primary neurons Human primary neurons (HPN), HEK293T Patient Cohort: 26 plasma samples of patients with AD, and 26 plasma samples of healthy control	circ_0002945↑/miR-431-5p↓/TNFAIP1↑/Aβ↑	Silencing circ_0002945 markedly attenuates neuronal apoptosis.	[140]
**hsa_circ_0007556**	Ex Vivo	Down-regulation	Patient Cohort: 29 postmortem human brain tissue diagnosed with AD, and 16 postmortem human brain tissue not diagnosed with dementia	circ_0007556↓/linear APP mRNA unchanged/Aβ-like peptide production	Its downregulation in the highly vulnerable entorhinal cortex inversely correlates with increased Aβ burden.	[119]
**circ-HUWE1**	In Vitro and Human serum samples	Up-regulation	In Vitro: SK-N-SH Patient Cohort: 33 plasma samples of patients with AD, and 20 plasma samples of healthy control	Aβ↑/circ-HUWE1↑/miR-433-3p↓/FGF7↑/Aβ↑	Silencing circ-HUWE1 significantly alleviates neuronal apoptosis and neuroinflammation	[136]
**hsa_circ_0049472**	In vitro, CSF and Human serum samples	Up-regulation	In Vitro: SK-N-SH, CHP-212 Patient Cohort: 20 plasma samples of patients with AD, and 20 plasma samples of healthy control	hsa_circ_0049472↑/miR-107↓/KIF1B↑/Aβ↑	Silencing circ_0049472 inhibits apoptosis, oxidative stress, and neuroinflammation.	[128]
**circ_0003611**	In Vitro and Human serum samples	Up-regulation	In Vitro: SK-N-SH Patient Cohort: 30 serum samples of patients with AD, and 20 serum samples of healthy control	circ_0003611↑/miR-885-5p↓/KREMEN1↑	Silencing circ_0003611 inhibits apoptosis, oxidative stress, and neuroinflammation.	[129]
**circ_0004381**	In Vitro and In Vivo	Up-regulation	In Vitro: Primary hippocampal neuron culture, BV2 cells. In Vivo: APP/PS1 mice, littermate mice	circ_0004381↑/miR-647↓/PSEN1↑/Aβ↑	High levels of circ_0004381 induces apoptosis, severe oxidative stress, mitochondrial dysfunction, and promotes M1-type microglial polarization	[30]
**circ_0003611**	In Vitro and Human Serum	Up-regulation	In Vitro: SH-SY5Y, SK-N-SH Patient Cohort: 36 serum samples of patients with AD, 36 serum samples of healthy controls	circ_0003611↑/miR-383-5p↓/KIF1B↑	Silencing of circ_0003611 diminishes apoptosis, oxidative stress, and restores glycolysis.	[130]
**circDlg1**	In Vitro, In Vivo and Ex Vivo	Up-regulation	In vitro: BV-2, HMC3, primary microglia (isolated from C57BL/6 mice), HEK293 Patient Cohort: Not specified	circDlg1↑/PDE4B↑/cAMP↓/Smurf2↑	Overexpression of circDlg1 causes decreased cognitive functions and increased neuroinflammation	[35]
**circ-Bptf**	In Vitro and and In Vivo	Down-regulation	In Vitro: HT22, HEK293 In Vivo: APP/PS1 (APPswe/PSEN1dE9) transgenic mice, WT C57BL/6	circ-Bptf↓/miR-138-5p↑/p62↓	Downregulation of circ-Bptf contributes to synaptic dysfunction and cognitive decline.	[32]

ZNF217: zinc finger protein 217; TNFAIP1: tumor necrosis factor, alpha induced protein 1; FGF7: fibroblast growth factor 7; KIF1B: kinesin family member 1B; KREMEN1: kringle containing transmembrane protein 1; Smurf2: SMAD specific E3 ubiquitin protein ligase 2.

## 6. Relationship Between Ferroptosis, Alzheimer’s Disease, and CircRNAs

### 6.1. Interactions Between Aβ, Tau, and Ferroptosis

**Figure 6:**
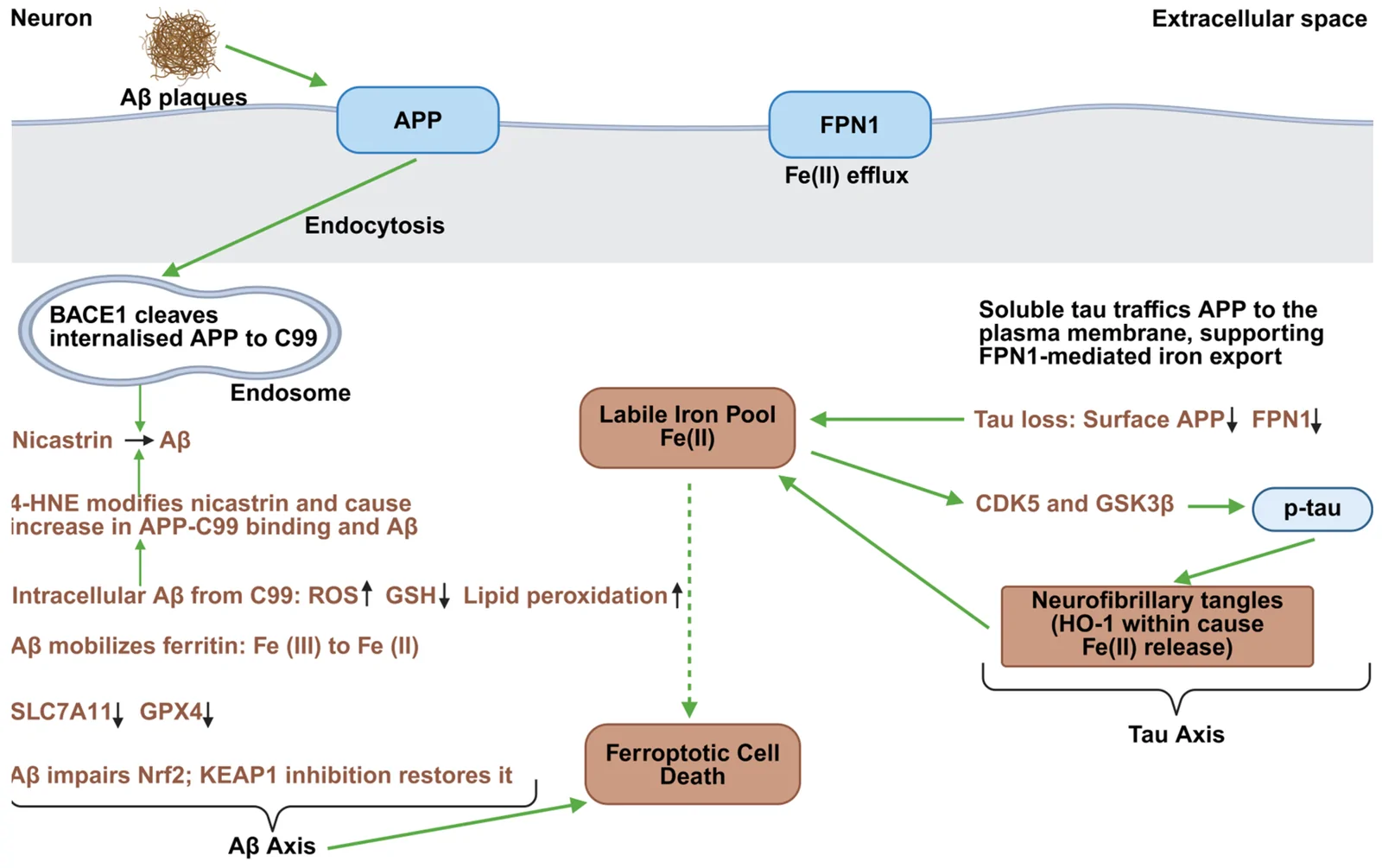
Interactions between amyloid-β, tau, neuronal iron homeostasis, and ferroptotic cell death in AD (Created in BioRender. Yuksel, M. (2026) https://BioRender.com/7obw520). Solid green arrows indicate established directional relationships, including activation, promotion, molecular traffick-ing, or progression between steps. The dashed green arrow indicates a proposed or indirect relationship. Upward (↑) and downward (↓) arrows indicate increased and decreased expression, activity, or abundance, respectively. Amyloidogenic processing of APP involves APP endocytosis followed by BACE1-mediated generation of C99 and subsequent γ-secretase-dependent production of Aβ [62]. Aβ-related processes may promote ferroptotic susceptibility through several mechanisms, including mobilisation of ferritin-bound iron, increased reactive oxygen species ROS and lipid peroxidation, GSH depletion, reduced SLC7A11 and GPX4 expression, and impairment of Nrf2-dependent antioxidant defence [141,142]. The lipid-peroxidation product 4-HNE can modify nicastrin, increasing its interaction with APP-C99 and thereby favouring Aβ generation, providing a potential link between lipid peroxidation and amyloidogenic processing [60,62]. Iron homeostasis also intersects with tau biology. Soluble tau facilitates trafficking of APP to the neuronal plasma membrane, where APP supports FPN1-mediated iron export; loss of soluble tau can therefore reduce surface APP and FPN1-dependent iron efflux, favouring expansion of the labile Fe(II) pool [49]. Increased intracellular iron and associated oxidative stress can promote tau phosphorylation through kinases including CDK5 and GSK3β, whereas iron associated with neurofibrillary pathology may further contribute to the intracellular labile iron pool [52]. These reciprocal interactions between amyloidogenic processing, tau dysfunction, iron accumulation, and lipid peroxidation may increase neuronal susceptibility to ferroptotic cell death [49,144].

Evidence from human AD brain tissue, patient-derived models, and experimental studies indicates a close relationship between amyloid pathology and ferroptosis mechanisms [141]. In post-mortem brain tissue from nine patients with AD and three controls, lower NCOA4 and GPX4 expression in grey matter correlated with increasing AD pathological severity. In the same study, patient-derived cerebral organoids developed Aβ-like aggregates accompanied by increased lipid peroxidation and altered iron homeostasis [141]. Treatment with the ferroptosis inhibitor ferrostatin-1 reduced lipid peroxidation, restored iron storage, and prevented Aβ-like aggregate formation [141] (Figure 6). These findings suggest that ferroptosis-related alterations in iron homeostasis and lipid peroxidation may contribute to Aβ pathology rather than occurring solely as downstream consequences of amyloid accumulation [141].

APP processing can directly influence neuronal iron homeostasis [142]. APP located at the neuronal plasma membrane stabilizes FPN1 and facilitates iron export [142] (Figure 6). During amyloidogenic processing, APP is internalised into endosomes, where it is cleaved by BACE1 [142]. This process decreases APP availability at the neuronal plasma membrane, resulting in less FPN1 stabilization, impaired iron export, and accumulation of intracellular labile iron [142]. The resulting decrease in FPN1 increases intracellular iron retention and expands the LIP, the redox-active fraction of intracellular iron (Figure 6) [142]. Inhibition of BACE1 or experimental prevention of APP endocytosis maintains APP at the neuronal surface, preserves FPN1 expression, and reduces intracellular iron accumulation [142]. These effects were predominantly observed in neurons and were influenced by AD-associated APP mutations, whereas Aβ itself and astrocytic APP had limited effects on this mechanism. Iron can also influence APP processing, as iron overload alters neuronal sAPPα trafficking and inhibits BACE1 activity [143] (Figure 6). These findings indicate a bidirectional relationship between APP processing and neuronal iron homeostasis [142,143].

Tau pathology is closely associated with neuronal iron homeostasis and ferroptosis in AD [49]. In addition to its role in microtubule stability, soluble tau contributes to neuronal iron export by facilitating the trafficking of APP to the plasma membrane, where APP stabilises FPN1 [49] (Figure 6). Tau deficiency therefore reduces APP and FPN1 on the neuronal membrane, resulting in neuronal iron accumulation and age-dependent neurodegeneration that can be attenuated by iron chelation [49]. These findings establish a functional relationship between soluble tau and neuronal iron export and suggest that loss of normal tau function during AD progression may contribute to iron accumulation [49].

Human neuropathological findings further link tau pathology, iron accumulation, and ferroptosis [145]. Neurons at an early stage of tau pathology, before the formation of mature neurofibrillary tangles, have been reported to undergo degeneration and fragment into droplet-like structures containing p-tau [145]. These structures are associated with iron accumulation and increased ferritin expression in surrounding microglia, indicating altered iron distribution during neuronal degeneration [145] (Figure 6). Based on these findings, Streit et al. (2022) proposed that this form of neuronal degeneration may involve ferroptosis [146]. Subsequent analysis of human AD brain tissue identified additional molecular changes associated with ferroptosis [147].

The regional association between iron and tau pathology has also been examined in vivo [148]. Quantitative susceptibility mapping (QSM), an MRI method, showed positive regional associations with tau-PET signal in individuals with AD, with the strongest correlations observed bilaterally in the inferior temporal gyrus (r = 0.31–0.32, *p* < 0.001) [148]. Further analysis indicated that iron burden partially accounted for the association between tau pathology and cortical atrophy, with a stronger effect in younger participants [148]. These findings may demonstrate a regional association among iron accumulation, tau pathology, and cortical neurodegeneration, although the observational design does not establish their causal sequence [148].

Oxidative stress may also promote tau phosphorylation through kinase activity [144]. In primary rat cortical neurons, combined copper chelation and Fe^2+^/H_2_O_2_-induced oxidative stress increased aberrant tau phosphorylation and GSK-3 activity compared with controls or treatment alone, whereas lithium reduced both effects [144] (Figure 6). Although ferroptosis was not directly assessed, these findings indicate that metal dyshomeostasis and oxidative stress can promote tau hyperphosphorylation through GSK-3 activation, suggesting a potential mechanism linking ferroptosis-associated oxidative stress to tau pathology [144].

### 6.2. Ferroptosis and Neuronal Inflammation

Ferroptosis and neuroinflammation are closely interconnected in AD through altered iron homeostasis, oxidative stress, and lipid peroxidation [62]. As discussed previously, lipid peroxidation products such as 4-HNE can also influence amyloidogenic APP processing, providing a link between ferroptotic stress and Aβ pathology. In addition, microglia and astrocytes can modify iron handling, inflammatory signalling, and antioxidant support, thereby influencing neuronal susceptibility to ferroptotic injury.

Microglia provide an important connection between neuroinflammation and ferroptosis because inflammatory activation alters their iron metabolism [149]. As described above, pro-inflammatory signalling can increase DMT1-dependent iron uptake and ferritin accumulation while reducing FPN1-mediated iron export, thereby increasing intracellular redox-active iron [144]. Experimental evidence further indicates that microglia can influence ferroptotic responses in other neural cells [15]. In human iPSC-derived cultures containing neurons, astrocytes, and microglia, iron overload produced the strongest transcriptional response in microglia and generated a ferroptosis-associated microglial population [15]. A genome-wide CRISPR screen identified SEC24B (SEC24 homolog B, COPII coat complex component) as a regulator of this response, while removal of microglia reduced neuronal lipid peroxidation and delayed neuronal death under ferroptotic conditions [15]. These findings indicate that microglial responses to iron can influence neuronal susceptibility to lipid peroxidation and ferroptotic injury.

**Figure 7:**
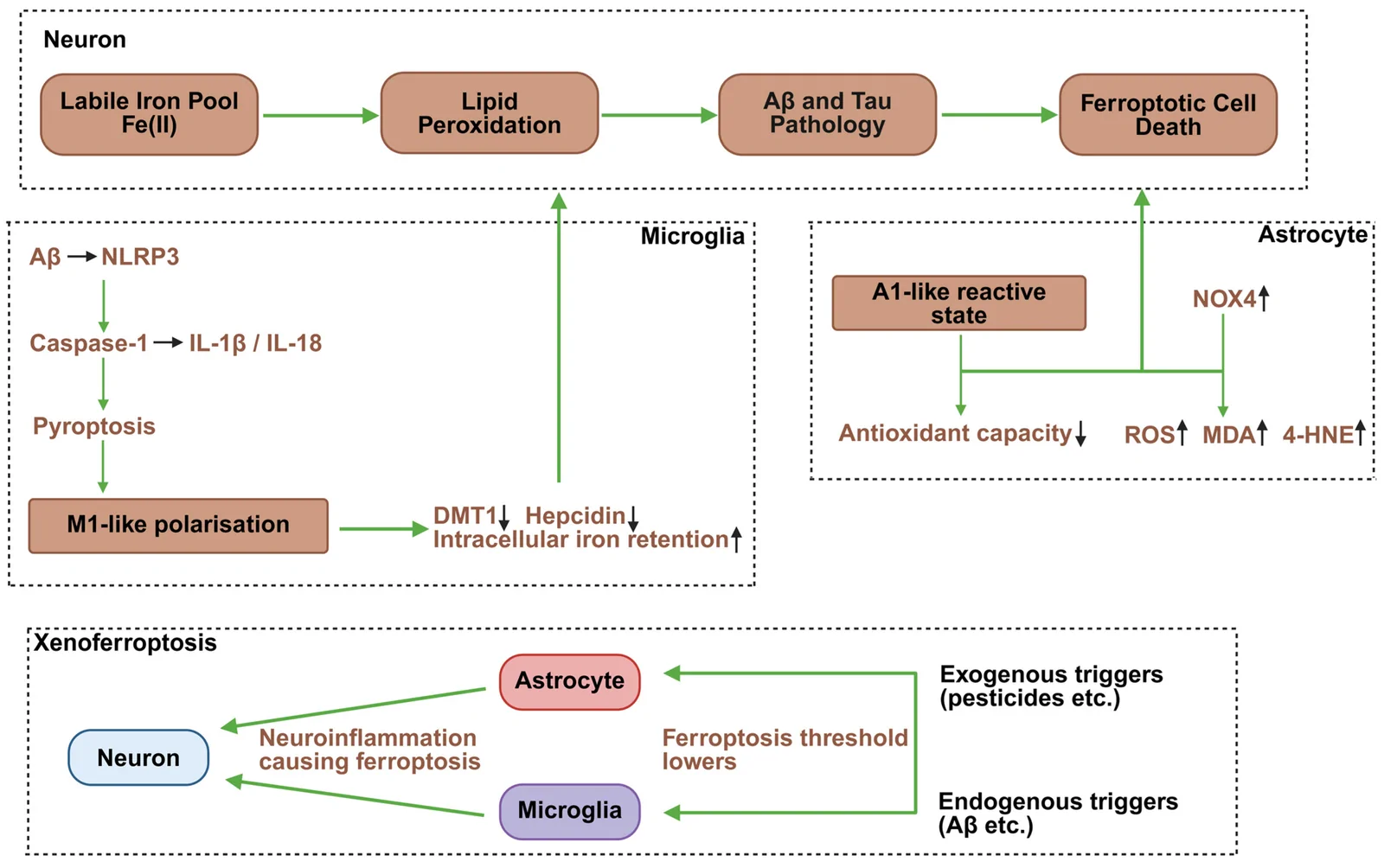
Interactions between neuroinflammation, glial dysfunction, and neuronal ferroptotic susceptibility in AD (Created in BioRender. Yuksel, M. (2026) https://BioRender.com/mhgswqu). Solid green arrows indicate directional relationships, including activation, promotion, progression, or transmission of effects between pathways and cell types. Branched arrows indicate that a common upstream process may influence more than one downstream target. ↑ and ↓ indicate increased and decreased expression, activity, or abundance, respectively. Neuronal ferroptosis is promoted by expansion of the labile Fe(II) pool and subsequent lipid peroxidation, which may interact with Aβ and tau pathology and ultimately contribute to ferroptotic cell death. Microglial activation provides a potential link between neuroinflammation and neuronal lipid peroxidation [149]. Aβ can activate the NLRP3 inflammasome, promoting caspase-1 activation, IL-1β and IL-18 release, pyroptosis, and pro-inflammatory M1-like microglial responses [155]. These states are accompanied by altered iron handling, including changes in DMT1 and hepcidin signalling and increased intracellular iron retention, which may enhance lipid-peroxidative stress in neurons [144,149]. Astrocytic dysfunction may further reduce neuronal resistance to ferroptotic injury. A1-like reactive states are associated with reduced antioxidant support, whereas increased NOX4 activity promotes ROS generation and accumulation of lipid-peroxidation products, including MDA and 4-HNE [150,152]. The lower panel illustrates the proposed concept of xenoferroptosis, in which endogenous triggers, including Aβ, or exogenous environmental stressors may alter astrocytic and microglial states, lower the threshold for ferroptosis, and promote neuroinflammatory conditions that increase neuronal ferroptotic susceptibility [154,156]. The relevance and molecular mechanisms of xenoferroptosis in AD remain to be established.

Astrocytes provide a complementary connection by supporting neuronal antioxidant defence [53,150]. They supply neurons with precursors required for glutathione synthesis and contribute to cerebral redox homeostasis [53,150]. During AD progression, astrocytes can acquire reactive states associated with increased inflammatory and neurotoxic activity [53,150]. In cortical astrocytes from patients with AD and APP/PS1 mice, increased NADPH oxidase 4 (NOX4) expression was accompanied by increased 4-HNE and MDA and impaired mitochondrial metabolism [151]. Although these changes do not establish ferroptosis in astrocytes, increased NOX4-derived ROS and lipid peroxidation, together with impaired astrocytic antioxidant support, could increase neuronal susceptibility to ferroptotic injury (Figure 7).

Genetic studies provide more direct evidence that disruption of ferroptosis-regulating pathways can cause neuronal degeneration and cognitive impairment in vivo [76]. Targeted deletion of GPX4 in mouse forebrain neurons increased lipid peroxidation and neuroinflammation and caused hippocampal neurodegeneration accompanied by spatial learning and memory deficits [76]. These effects were accelerated by vitamin E deficiency and attenuated by pharmacological inhibition of ferroptosis [76]. Because this model does not reproduce Aβ or tau pathology, it demonstrates that disruption of GPX4-mediated protection against lipid peroxidation can independently induce neuronal degeneration and cognitive impairment [76] (Figure 7). Similarly, neuronal deletion of FPN1 caused intracellular iron accumulation, increased MDA, reduced GSH, and ferroptosis-associated neuronal death [152]. APP/PS1 mice showed related alterations in iron accumulation, lipid peroxidation, and ferroptosis-associated gene expression, with transcriptomic analysis identifying overlapping changes in ferroptosis- and AD-associated pathways [65,152]. Together, these models support a mechanistic relationship between impaired iron export or antioxidant defence and neuronal ferroptotic injury [76,153].

Ferroptosis-related damage may also involve interactions between neural cell populations. The recently proposed concept of xenoferroptosis describes intercellular propagation of ferroptosis-associated stress following exposure to exogenous factors or endogenous factors such as Aβ oligomers [154] (Figure 7). Its relevance and underlying mechanisms in AD remain to be established, but the findings described above support investigating ferroptosis in multicellular rather than exclusively neuron-autonomous models. iPSC-derived co-cultures and brain organoids may therefore help determine how neuronal, microglial, and astrocytic responses interact during ferroptotic stress [38].

### 6.3. CircRNAs as Candidate Regulators of Ferroptosis in Alzheimer’s Disease

Ferroptosis is associated with Aβ and tau pathology, neuronal degeneration, and glial inflammatory responses in AD. CircRNAs have also emerged as regulators of ferroptosis through their effects on iron homeostasis, antioxidant defence, and lipid metabolism. Although these mechanisms have been demonstrated in several experimental disease models, direct evidence that circRNAs regulate ferroptosis in AD remains limited.

In high-glucose-treated retinal pigment epithelial cells, circPSEN1 promotes ferroptosis through the miR-200b-3p/CFL2 (cofilin-2) axis [157]. Silencing circPSEN1 increases miR-200b-3p activity and reduces CFL2 expression, accompanied by increased GSH, GPX4, and SLC7A11, reduced intracellular Fe^2+^ and MDA, and decreased cell death [151]. In hepatocellular carcinoma cells, cIARS regulates ferroptosis through a different mechanism involving ferritinophagy [158]. cIARS interacts with the m6A demethylase ALKBH5 (AlkB homolog 5, RNA demethylase) and suppresses its inhibitory effect on autophagy, thereby promoting autophagic flux and ferritinophagy and increasing sensitivity to sorafenib- and erastin-induced ferroptosis [159]. These studies demonstrate that circRNAs can regulate ferroptosis through both miRNA-dependent regulation and interactions with RBPs, but whether comparable mechanisms occur in AD remains to be established [157,158].

Evidence from brain-derived cells further demonstrates that circRNAs can regulate ferroptosis. In glioma cells, circCDK14 reduces sensitivity to erastin-induced ferroptosis through the miR-3938/PDGFRA (platelet-derived growth factor receptor alpha) axis [159]. CircCDK14 sponges miR-3938, thereby increasing PDGFRA expression, while manipulation of this pathway alters intracellular iron and ROS levels and the mitochondrial morphological changes associated with ferroptosis [159]. These findings can provide evidence that a circRNA can regulate susceptibility to ferroptosis in brain-derived cells through a defined miRNA–mRNA pathway. However, this mechanism was identified in glioma cells, and whether circRNAs similarly regulate ferroptosis in neurons or glial cells during AD remains unknown.

CircRNAs may also influence ferroptosis indirectly by regulating microglial inflammatory states. As discussed above, the M1-like phenotype is associated with increased iron uptake and retention, ROS production, and lipid peroxidation, all of which can increase susceptibility to ferroptosis [15,149]. In AD-related models, the circAPP/miR-1906/CLIC1 axis suggests that silencing of circAPP can improve cognitive function by shifting microglia toward an M2-like phenotype, as discussed earlier [33]. Conversely, mmu_circ_0000842/miR-770-3p/TREM2 indicates that mmu_circ_0000842 expression decreases glial activity, as explained previously [99]. Although neither study assessed ferroptosis, these findings identify circRNA-regulated microglial pathways that could influence ferroptosis susceptibility through changes in inflammatory state, iron metabolism, and oxidative stress. Direct measurement of iron accumulation, lipid peroxidation, GPX4 activity, and ferroptotic cell death will be required to determine whether this relationship occurs in AD.

#### The cAMP-EPAC Axis as Isoform Selective Entry Point

**Figure 8:**
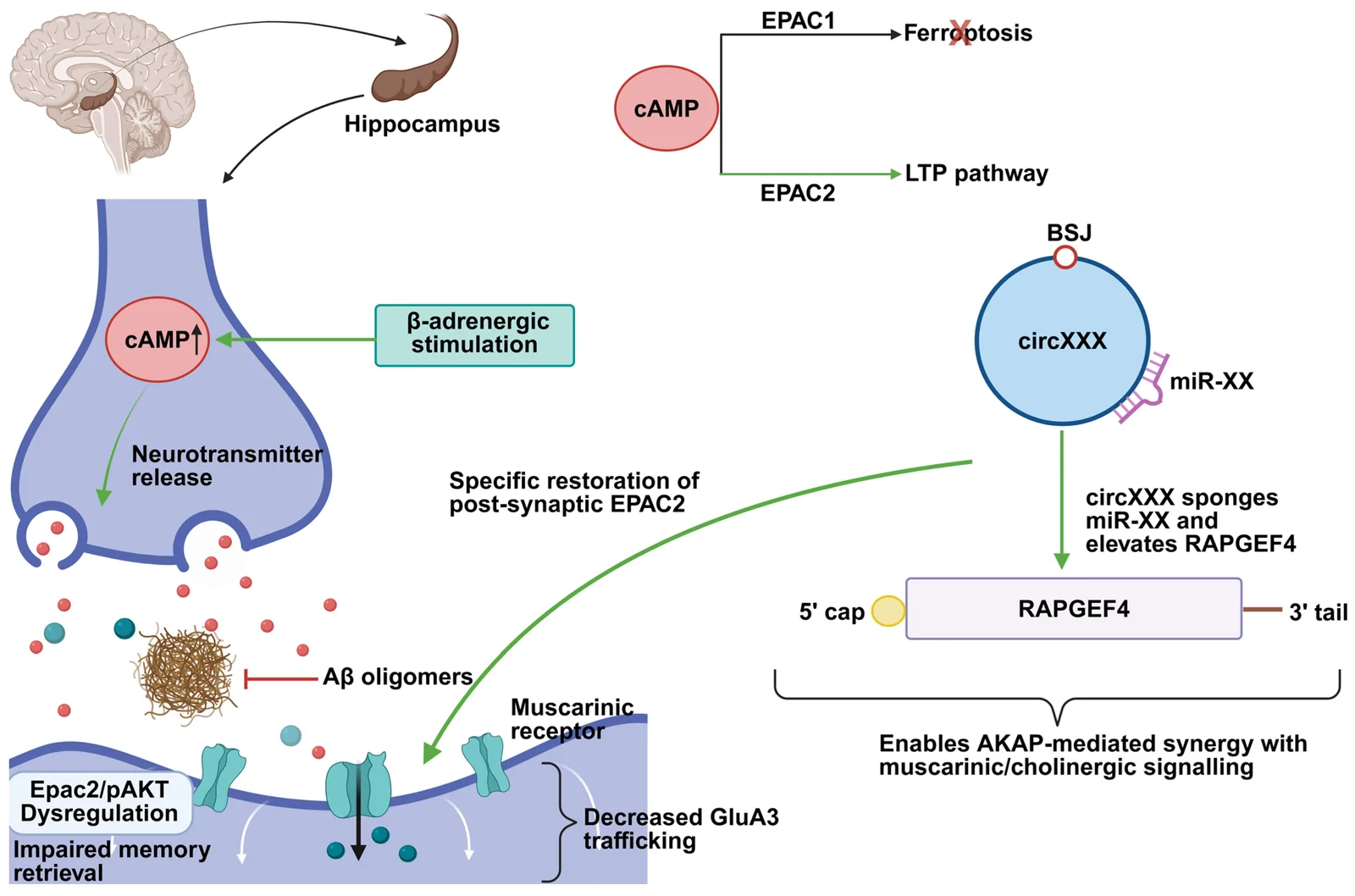
Proposed therapeutic targeting of the circRNA–RAPGEF4/EPAC2 axis to restore synaptic plasticity and memory retrieval in AD (Created in BioRender. Yuksel, M. (2026) https://BioRender.com/1fv3iku). Solid arrows indicate the direction of signalling, activation, regulation, or pathway progression. The red blunt-ended line indicates inhibition. ↑ indicates increased expression or activity. Under physiological conditions, β-adrenergic stimulation produces localised increases in cAMP within synaptic compartments, supporting neurotransmission and activation of postsynaptic cAMP effectors [162]. EPAC2/RAPGEF4 contributes to postsynaptic plasticity by promoting trafficking and surface incorporation of GluA3-containing AMPA receptors, thereby supporting LTP and memory retrieval [68]. In AD, soluble Aβ oligomers disrupt this signalling network and are associated with reduced or dysregulated EPAC2 and pAKT signalling, impaired GluA3 trafficking, and deficits in memory retrieval [123]. The upper schematic illustrates the functional distinction between EPAC isoforms: EPAC2 is predominantly associated with neuronal synaptic regulation and LTP-related signalling, whereas EPAC1 has been implicated in ferroptosis-related pathways [156]. These observations provide a rationale for selectively restoring EPAC2-associated signalling rather than globally increasing cAMP, which would activate multiple downstream effectors. The right-hand pathway depicts a hypothetical circRNA-based strategy for increasing RAPGEF4 expression. An endogenous or engineered circRNA containing a defined BSJ and miRNA-binding sites could sequester a RAPGEF4-targeting miRNA, thereby reducing miRNA-mediated repression of RAPGEF4 and increasing EPAC2 expression. Such an approach could, in principle, provide greater transcript-level selectivity than non-selective pharmacological elevation of cAMP, although the specific circRNA–miRNA pair represented as circXXX/miR-XX has not yet been established in AD neurons. Selective restoration of EPAC2 could promote GluA3-containing AMPAR trafficking and improve postsynaptic plasticity without directly increasing EPAC1 activity. EPAC2 restoration may also interact with other postsynaptic signalling systems. Within AKAP-organised signalling complexes, cAMP-dependent pathways can function alongside muscarinic/cholinergic signalling, suggesting a potential mechanism through which EPAC2-directed interventions could enhance receptor trafficking and synaptic responsiveness. Whether circRNA-mediated restoration of RAPGEF4 can produce such effects, or augment the actions of cholinergic therapies, remains to be tested experimentally. Solid arrows indicate established or proposed stimulatory relationships shown in the model, inhibitory bars indicate suppression, and the circXXX/miR-XX/RAPGEF4 pathway represents a proposed therapeutic mechanism rather than an experimentally validated AD pathway.

cAMP signalling regulates several cell-death pathways implicated in AD, including parthanatos, lysosome-dependent cell death, senescence, and ferroptosis [5]. Its effects are mediated through downstream effectors including PKA and EPACs [127]. In ferroptosis, PKA signalling has been associated with protection against lipid peroxidation, whereas EPAC1, encoded by RAPGEF3 (Rap guanine nucleotide exchange factor 3), can promote ferroptotic responses [127]. EPAC2, encoded by RAPGEF4 (4), has distinct functions in the brain, including regulation of synaptic plasticity and neuronal survival [127]. These differences suggest that the relative regulation of EPAC1 and EPAC2 may be relevant to the relationship between cAMP signalling, ferroptosis, and neuronal dysfunction in AD. However, their distinct roles in neuronal ferroptosis have not yet been established (Figure 8).

CircRNAs may provide a mechanism for the selective regulation of EPAC signalling at the transcript level. CDR1as/ciRS-7, a highly expressed brain circRNA containing multiple miR-7-binding sites, is decreased in AD brain tissue [160], while dysregulation of the CDR1as/miR-7 pathway has also been implicated in AD-related molecular changes [95]. Independently, miR-7 has been shown to regulate EPAC1/RAPGEF3 in non-neuronal cells [94]. These findings raise the possibility of a CDR1as/miR-7/EPAC1 regulatory axis in AD, but this complete pathway has not been experimentally demonstrated. Its relevance to ferroptosis is also unknown. Future studies should therefore determine whether altered CDR1as expression changes miR-7 availability and EPAC1 expression in AD neurons and whether these changes affect lipid peroxidation and susceptibility to ferroptosis.

A separate possibility involves EPAC2. Selective circRNA-mediated regulation of RAPGEF4 could potentially modify EPAC2-dependent neuronal signalling without simultaneously activating EPAC1. EPAC2 participates in the trafficking of GluA3-containing AMPA receptors and memory-related synaptic plasticity [161], but no circRNA regulating RAPGEF4 in AD neurons has yet been identified. Identification of such circRNAs, followed by validation in neuronal models containing AD-related amyloid and tau pathology, would be required to determine whether selective regulation of EPAC1 or EPAC2 can simultaneously influence ferroptosis and synaptic dysfunction (Figure 8).

## 7. Discussion and Outlook

CircRNAs have emerged as potential regulators and biomarkers in AD through diverse mechanisms, including miRNA sponging, interactions with RBPs, regulation of protein degradation, and translation [20,21]. AD-associated circRNAs have been linked mainly to amyloidogenic APP processing, neuroinflammation, and synaptic dysfunction, whereas their relationships with tau pathology and ferroptosis remain much less defined. Although ferroptosis is increasingly implicated in AD through iron dysregulation, lipid peroxidation, and impaired antioxidant defence, direct circRNA–ferroptosis mechanisms have not yet been demonstrated in AD.

The mechanisms discussed in this review should not be considered independently. Amyloidogenic APP processing, tau dysfunction, neuroinflammation, synaptic impairment, and ferroptosis are interconnected through mechanisms involving iron homeostasis, oxidative stress, lipid peroxidation, and protein regulation. CircRNA dysregulation may therefore affect several interconnected AD-related processes rather than a single pathway. However, current evidence is limited by small clinical cohorts, reliance on simplified cellular models, and incomplete validation in human-derived systems. The following sections focus on the major methodological and mechanistic gaps that currently limit the diagnostic and therapeutic interpretation of AD-associated circRNAs.

### 7.1. Limitations of circRNAs as Biomarkers in Alzheimer’s Disease

Several limitations need to be addressed before circulating circRNAs can be reliably used as clinical biomarkers in AD. Current evidence is based on a small number of patient cohorts, and independent validation remains limited. In some studies, discovery and validation were performed within the same overall dataset or study framework, which may overestimate diagnostic performance (Table 1). Reported AUC values therefore cannot be compared directly across studies because of differences in sample type, cohort composition, analytical methods and study design. Sex-stratified analyses are also inconsistently reported, further limiting assessment of whether biomarker performance differs between females and males (Table 1).

Analytical standardization represents another challenge. CircRNA quantification lacks universally accepted reference transcripts, and differences in normalisation, pre-analytical processing, primer design and assay conditions may contribute to variability between studies [163]. ddPCR (digital droplet PCR) may support validation by enabling absolute quantification without a standard curve, but it cannot overcome variability arising from sample handling, assay design or normalisation. Standardized analytical workflows will therefore be necessary for reproducible clinical evaluation.

The biological origin and functional relevance of circulating circRNAs remain uncertain. Detection of a circRNA in peripheral blood does not show that it originates from the brain or that its circulating level reflects changes within affected brain regions, particularly when its proposed function has been studied only in neural tissue or experimental models. Another limitation is that diagnostic and mechanistic studies often focus on different circRNAs (Table 1 and Table 2). Many biomarker candidates have not been functionally characterised, while circRNAs with demonstrated roles in AD-related pathways have rarely been evaluated for diagnostic performance. In addition, the function of a circRNA should not be assumed based only on its parental gene. Studies comparing circRNA levels in brain tissue, extracellular vesicles, and peripheral samples may help determine whether circulating circRNAs reflect changes occurring in the AD brain.

The clinical value of circRNAs should also be assessed together with established AD biomarkers. Plasma p-tau217 and Aβ42/40 already provide diagnostic and prognostic information [37], whereas circRNA candidates have not been evaluated together with these markers. It therefore remains unclear whether circRNAs improve diagnostic or prognostic assessment when combined with established amyloid- and tau-based biomarkers. Their clinical purpose should also be clearly defined, as biomarkers for diagnosis, prognosis, disease monitoring, or pathological burden require different forms of validation. Larger independent cohorts, standardised measurement, and direct comparison with established biomarkers will be needed to determine the clinical usefulness of circulating circRNAs in AD.

### 7.2. CircRNAs Regulate Amyloidogenic Mechanism

The findings discussed in Section 5.1 suggest that individual circRNAs may influence more than one process involved in Aβ production and clearance. However, it is not always clear whether the reported effects represent multiple actions of the same circRNA or differences between experimental models. This is particularly important for ceRNA mechanisms, which depend on the relative abundance and localisation of the circRNA, miRNA, and target mRNA. For example, circCwc27 affects both Aβ production and clearance [27], indicating that a single circRNA may influence several processes involved in Aβ homeostasis. circ_0003611 has also been linked to two different pathways, miR-885-5p/KREMEN1 and miR-383-5p/KIF1B [129,130] (Table 2). However, these studies do not show that both interactions occur simultaneously in the same cells. The reported differences may therefore reflect multiple functions of circ_0003611, but may also result from differences in cell type, experimental conditions, or miRNA abundance.

Current studies often examine individual circRNA–miRNA–mRNA pathways separately. Future studies should determine whether these interactions occur simultaneously within the same cell type and assess their relative contribution to Aβ pathology.

### 7.3. Synaptic Dysfunction and the cAMP–EPAC Axis

Synaptic dysfunction has been linked to circRNA dysregulation in AD, particularly through circRIMS2, which affects GluN2B stability, dendritic spine density, and memory [31]. However, current evidence remains focused on individual receptor-associated pathways. An important question is whether circRNAs also influence broader signalling networks that coordinate multiple components of synaptic plasticity.

The cAMP–EPAC pathway may represent a potential connection between circRNA dysregulation, synaptic dysfunction, and ferroptosis in AD. circ-Dlg1 and ciRS-7 are associated with cAMP-related signalling [35,95,125], although direct circRNA regulation of EPAC signalling has not been demonstrated in AD neurons. It may be particularly relevant because EPAC2 is involved in receptor trafficking and synaptic plasticity [161], whereas EPAC1 has been implicated in ferroptosis-related signalling [161]. These findings do not establish opposing functions for the two isoforms or an anti-ferroptotic role for EPAC2, but suggest that circRNA-dependent alterations in cAMP signalling may differentially affect EPAC-associated synaptic and ferroptosis-related processes. Direct assessment of EPAC1 and EPAC2 following circRNA manipulation will therefore be important to determine whether circRNAs influence these pathways in AD-relevant neuronal models.

Upstream regulation of circRIMS2 may also influence its synaptic effects. METTL3-dependent m6A modification contributes to increased circRIMS2 abundance in AD models [31], suggesting that altered RNA modification may affect synaptic function indirectly by changing circRIMS2 levels. However, whether modulation of METTL3 alters GluN2B stability or other circRIMS2-dependent synaptic outcomes has not been established.

### 7.4. CircRNA Regulates Neuroinflammation

CircRNA studies in AD models indicate that microglial inflammatory and phagocytic functions can be modified through several regulatory pathways, including those involving circAPP, circ_0061183, and exosomal circ-Epc1 [33,34,99]. However, these findings are commonly interpreted using the conventional M1/M2 framework, which does not adequately reflect the heterogeneity of microglial states in AD [13]. Changes in Aβ phagocytosis, inflammatory signalling, and cognition may therefore better reflect the biological effects of individual circRNAs than changes in M1- or M2-associated markers alone. Future studies should combine cell-type-resolved molecular profiling with functional measurements to determine which microglial states and functions are affected by individual circRNAs.

An important unresolved question is whether AD-associated circRNAs also influence inflammasome activation and pyroptosis. Aβ-associated microglial responses can involve NLRP3 activation, caspase-1-dependent maturation of IL-1β and IL-18, gasdermin-mediated pyroptosis, and altered iron metabolism [54], but these processes have not been directly examined for the circRNAs discussed in this review. This distinction is important because commonly measured endpoints such as ROS production, cytokine release, and cell viability are shared by several forms of cellular stress and death. Future studies should distinguish pyroptosis from ferroptosis using specific molecular markers. Ferroptosis should also be confirmed by iron-dependent lipid peroxidation and response to ferroptosis inhibitors.

Astrocytic circRNA regulation remains even less defined. Astrocytes participate in inflammatory, redox, and metabolic processes relevant to AD, including RAGE/NOX-dependent ROS generation, Nrf2/NF-κB signalling, and lipid peroxidation [151,164,165]. However, current evidence does not establish direct regulation of these processes by AD-associated circRNAs in astrocytes. Although circCwc27 silencing reduces astrocytic as well as microglial activation [27], this finding does not distinguish a cell-autonomous astrocytic effect from a secondary response to changes in other cell types. Future studies should test whether candidate circRNAs directly affect astrocyte function, particularly inflammatory signalling, antioxidant defence, iron handling, and lipid peroxidation.

### 7.5. CircRNAs at the Interface of Tau Dysfunction and Ferroptosis in AD

#### 7.5.1. The Underexplored circRNA–Tau Relationship

CircRNA regulation of tau remains substantially less characterized than circRNA regulation of Aβ pathology. None of the circRNAs discussed in Table 2 have been directly shown to regulate tau phosphorylation, aggregation, clearance, or soluble tau levels. However, several are linked to pathways involved in tau regulation and therefore provide candidates for direct experimental investigation.

circDlg1 regulates cAMP signalling through the PDE4B/cAMP/PKA/CREB pathway [35], while ciRS-7 may influence EPAC-related signalling through miR-7 [95,125]. Their effects on tau have not been examined, and the reported changes in ciRS-7 and EPAC1 in AD do not support a simple regulatory relationship [123]. Assessing tau phosphorylation, abundance, and turnover following circDlg1 or ciRS-7 manipulation could therefore determine whether circRNA-dependent alterations in cAMP–EPAC signalling extend to tau-related mechanisms.

Protein homeostasis provides another potential connection. circ-Bptf, circRIMS2, and ciRS-7 regulate pathways associated with autophagic or ubiquitin-dependent protein handling [25,26,31,32], but whether these effects influence tau degradation remains unknown. Future studies should determine whether these circRNAs alter soluble, phosphorylated, or aggregated tau.

Tau-dependent regulation of mitochondrial function may provide an additional link. Aβ oligomers disrupt lysosomal mTORC1-dependent control of mitochondrial activity through a tau-dependent mechanism [134]. circ_0004381 is relevant in this context because its manipulation affects mitochondrial dysfunction and oxidative stress [30], although there is currently no evidence that these effects involve tau. Testing circ_0004381 and other mitochondria-associated circRNAs in models containing both Aβ and tau pathology could therefore clarify whether circRNA-dependent mitochondrial changes are connected to tau dysfunction.

#### 7.5.2. Potential Links Between AD-Associated circRNAs and Ferroptosis

Several AD-associated circRNAs regulate pathways that could influence ferroptotic susceptibility, although these relationships have not been directly tested. circCwc27 and ciRS-7 regulate APP-related processes [26,27], raising the possibility that they may also affect surface APP/FPN1-dependent neuronal iron export. This could be tested by measuring surface APP, FPN1 and intracellular iron following circRNA manipulation (Table 3).

circ_0004381 provides another candidate because it regulates PSEN1 [30], while PSEN signalling has independently been linked to GPX4-dependent neuronal antioxidant defence [78]. Whether circ_0004381-mediated changes in PSEN1 influence GPX4 activity, lipid peroxidation or ferroptotic susceptibility requires direct investigation.

circAPP and circ_0061183 modify microglial responses, while changes in microglial iron uptake, storage, and inflammatory activity can influence ferroptotic responses in neurons [33,34]. Cell-type-specific manipulation in multicellular models could therefore determine whether glial circRNAs alter neuronal ferroptotic susceptibility indirectly through changes in iron homeostasis or inflammatory signalling.

Together, these candidates provide testable links between AD-associated circRNAs and ferroptosis, but none currently establishes a circRNA–ferroptosis pathway in AD. Additional potential relationships are summarized in Table 3.

**Table 3:** Hypothetical links among circRNAs and ferroptosis in AD.

CircRNA Name	Expression Pattern in AD	Established AD Pathway	Proposed Ferroptosis Node	Hypothetical circRNA/AD/Ferroptosis Link	Ref.
AD-Associated CircRNAs with Proposed Ferroptosis Links					
circCwc27	Up-regulation	circCwc27↑/Pur-α↓/Aβ↑	Iron homeostasis	Surface APP?/FPN1↓?/LIP↑?/lipid peroxidation↑?	[27]
ciRS-7/CDR1as	Down-regulation	ciRS-7↓/miR-7↑/UBE2A↓ ciRS-7↓/NF-κB/UCHL1↑/APP↑/BACE1↑	cAMP–EPAC axis	miR-7↑/EPAC1↓?/lipid peroxidation?	[95,125]
circ_0004381	Up-regulation	circ_0004381↑/miR-647↓/PSEN1↑/Aβ↑	Antioxidant defence (Notch-1/LRP8/selenium/GPX4)	PSEN1↑/LRP8?/GPX4↓?/lipid peroxidation↑?	[30]
circAPP	Up-regulation	circAPP↑/miR-1906↓/CLIC1↑/Aβ↑	Glia-mediated	M1↑/DMT1↑/FPN1↓/microglial iron↑?/neuronal lipid peroxidation↑?	[33]
circ_0061183	Down-regulation	circ_0061183↓/miR-98-5p↑/Aβ↓	Glia-mediated	M1↑/iron retention↑?/neuronal ferroptosis↑?	[34]
circ-Epc1	Down-regulation	circ-Epc1↑/miR-770-3p↓/TREM2↑/M2↑	Glia-mediated	M2↑/microglial iron retention↓?/neuronal ferroptosis↓?	[99]
circDlg1	Up-regulation	circDlg1↑/PDE4B↑/cAMP↓/Smurf2↑	cAMP–EPAC axis	cAMP↓/PKA↓ (lipid-peroxidation protection↓?)/EPAC1?/EPAC2?; tau phosphorylation?	[35]
circRIMS2	Up-regulation	circRIMS2↑/miR-3968↓/UBE2K ↑/GluN2B↓	Excitotoxicity-coupled ferroptosis	NMDAR dysregulation/Ca^2+^↑/ROS↑/ferroptosis?	[31]
circ-Bptf	Down-regulation	circ-Bptf↓/miR-138-5p↑/p62↓	Ferritinophagy (NCOA4/ferritin → LIP)	p62↓/ferritinophagy?/LIP↑?	[32]
circLPAR1	Up-regulation	circLPAR1↑/miR-212-3p↓/ZNF217 ↑/Aβ↑	Unassigned oxidative/cell-death phenotype	ROS↑/viability↓/ferroptosis↑?	[118]
hsa_circ_0049472	Up-regulation	hsa_circ_0049472↑/miR-107↓/KIF1B↑/Aβ↑	Unassigned oxidative/cell-death phenotype	ROS↑/viability↓/ferroptosis↑?	[126]
CircRNAs with Demonstrated Ferroptosis Regulation in Other Diseases					
circPSEN1	Up-regulation (retinal pigment epithelial cells)	circPSEN1↑/miR-200b-3p↓/CFL2↑/GPX4↓/GSH↓/SLC7A11↓/Fe^2+^↑/MDA↑	Antioxidant defence (GPX4/GSH/SLC7A11)	PSEN/Notch-1/LRP8/GPX4 in AD neurons?	[157]
cIARS	Up-regulation (hepatocellular carcinoma)	cIARS/ALKBH5↓/autophagy↑/ferritinophagy↑/ferroptosis sensitivity↑	Ferritinophagy (NCOA4/ferritin → LIP)	RBP-mediated, not ceRNA; NCOA4/ApoE–PI3K/Akt relevance in AD?	[158]
circCDK14	Up-regulation (glioma cells)	circCDK14↑/miR-3938↓/PDGFRA↑/erastin sensitivity↓/iron, ROS altered	Iron homeostasis and lipid peroxidation	Proof of principle in brain-derived cells; neurons/glia in AD?	[159]
Engineered/Proposed CircRNA-Ferroptosis Intervention					
circXXX/miR-XX (proposed)	Engineered/restored	circXXX/miR-XX↓/RAPGEF4↑/EPAC2↑/GluA3-AMPAR↑/LTP↑	EPAC2-selective restoration while avoiding EPAC1-associated ferroptosis	Transcript-level selectivity vs. non-selective cAMP elevation	

#### 7.5.3. The circRNA–Ferroptosis Evidence Gap

As discussed previously, commonly used endpoints in AD circRNA studies are not sufficient to establish ferroptosis, and direct ferroptosis-specific validation remains largely absent.

Evidence from non-AD systems nevertheless shows that circRNAs can regulate ferroptosis through distinct mechanisms. circPSEN1 has been linked to ferroptosis-related changes through the miR-200b-3p/CFL2 pathway in high-glucose-treated retinal pigment epithelial cells [157], cIARS regulates ferritinophagy through ALKBH5-dependent autophagic mechanisms in hepatocellular carcinoma [158], and circCDK14 modifies ferroptotic susceptibility through the miR-3938/PDGFRA pathway in glioma cells [159]. These studies suggest that circRNAs can regulate ferroptosis in other disease models, but whether the same mechanisms operate in AD-related neural cells remains unknown.

circPSEN1 is particularly relevant for further investigation because it is derived from a gene directly implicated in familial AD and has already been associated with ferroptosis-related changes in another cellular context [157,166]. PSEN signalling is also independently linked to neuronal antioxidant defence through Notch-1/LRP8-dependent selenium uptake and GPX4 synthesis [74]. These findings do not establish a circPSEN1–GPX4 pathway, but they may provide a specific rationale for testing whether circPSEN1 influences ferroptotic susceptibility in AD-relevant neural models (Table 3).

The relationship between circRNAs and ferroptosis may also involve tau-related mechanisms. Soluble tau contributes to neuronal iron export, whereas iron accumulation can promote tau phosphorylation and increase susceptibility to lipid peroxidation. Experiments that assess tau together with intracellular iron, lipid peroxidation, GPX4-related defence, and ferroptosis-specific rescue could therefore address both questions within the same model. This would help determine whether circRNA-dependent alterations in tau and iron homeostasis contribute to ferroptotic susceptibility in AD.

### 7.6. Therapeutic Considerations and Limitations of circRNA-Directed Intervention in Alzheimer’s Disease

The therapeutic potential of circRNAs in AD depends not only on whether individual circRNAs modify disease-associated pathways but also on whether they can be manipulated selectively, delivered to the relevant cells, and evaluated in models that adequately represent human disease. The available evidence identifies several distinct challenges involving target selection, biological context, delivery, experimental models, and therapeutic assessment. These issues should be considered separately because some represent current technical limitations, whereas others define requirements for the development and validation of circRNA-directed interventions.

#### 7.6.1. Biological Context and Pathway Interactions

The effects of circRNA manipulation may depend strongly on pathological context. ciRS-7 illustrates this clearly. In AD, reduced ciRS-7 has been associated with impaired UBE2A-dependent Aβ clearance [25], whereas in ischaemic models, reduced ciRS-7 increases miR-7 availability and can attenuate excitotoxic neuronal injury [95]. The same direction of circRNA manipulation may therefore have different effects under different disease conditions, indicating that individual circRNAs should not be considered uniformly beneficial or detrimental.

Therapeutic effects may also extend beyond a single downstream pathway. Because individual circRNAs can influence several targets, manipulation of one circRNA may produce multiple molecular and functional effects. Therapeutic studies should therefore evaluate the main downstream pathways relevant to the proposed mechanism rather than attributing the observed effect to a single interaction alone.

#### 7.6.2. Anatomical Specificity and CNS Delivery

The therapeutic approach will depend on the direction and functional consequences of circRNA dysregulation. CircRNAs that are increased and associated with detrimental effects may require suppression, whereas reduced circRNAs associated with beneficial effects may require replacement or restoration. BSJ-directed antisense oligonucleotides or RNA-targeting systems such as Cas13 could provide sequence-specific suppression, while restoration would require delivery of the circRNA itself or sustained expression from an appropriate vector. These approaches differ in reversibility, duration of effect, and control of circRNA abundance.

A further challenge is achieving sufficient exposure in the relevant CNS cell populations while limiting effects in peripheral tissues. Sequence-specific recognition does not provide anatomical or cell-type specificity, and the same circRNA may have different functions across tissues. Delivery across the blood–brain barrier will therefore require approaches capable of reaching the relevant neuronal or glial populations. Potential strategies include engineered extracellular vesicles, CNS-targeted lipid nanoparticles, and viral vectors with appropriate cellular tropism. Exosomal delivery of circ-Epc1 has produced beneficial effects in experimental models [99], demonstrating the feasibility of exogenous circRNA delivery, although this does not establish restoration of endogenous circRNA regulation in the AD brain.

The required duration of treatment will also depend on the therapeutic strategy. Suppression may be achieved with transient and reversible approaches, whereas restoring a reduced circRNA may require repeated RNA delivery or sustained expression. Because the physiological expression range of many AD-associated circRNAs remains poorly defined, dose, duration, and tissue distribution will need to be controlled carefully. Manipulation of upstream RNA-modification machinery should also be distinguished from direct circRNA targeting, as it may affect numerous circular and linear transcripts rather than a single circRNA.

#### 7.6.3. Safety of circRNA Restoration and RNA Modification

Restoration of downregulated circRNAs requires careful control of expression level and duration, particularly because some circRNAs can undergo cap-independent translation [93,125]. Therapeutic replacement may therefore generate protein products in addition to restoring the intended RNA-mediated function, especially when circRNAs are expressed above endogenous levels or through sustained vector-mediated delivery. Exogenous delivery should also be distinguished from restoration of endogenous circRNA regulation, as increasing circRNA abundance does not necessarily reproduce its normal expression pattern or biological state.

circMAN2A1 provides an AD-specific example of this concern. A-to-I editing alters its coding potential and enables production of chimeric peptides associated with AD progression [28]. Although this mechanism cannot be generalised to other circRNAs, it shows that circRNA abundance alone may not predict biological output. Candidate circRNAs intended for replacement should therefore be assessed for coding potential, editing state, and possible translation products before sustained therapeutic expression is considered.

RNA modification introduces further complexity. ADAR-dependent editing can influence circRNA biogenesis and, in specific cases, coding potential [28,90], whereas m6A can affect circRNA abundance or translation depending on the transcript and cellular context [31,94,96]. Manipulating these regulatory mechanisms may therefore alter several properties of a circRNA simultaneously. Therapeutic evaluation should consequently assess circRNA abundance, sequence modification, and translational output to determine whether replacement reproduces the intended function without introducing additional effects.

#### 7.6.4. Timing and Assessment of Therapeutic Effects

The timing of circRNA-directed intervention may influence therapeutic efficacy. Current disease-modifying therapies for AD generally show greater effects when administered during earlier, biomarker-confirmed disease, and lower baseline tau burden has been associated with greater treatment effects in donanemab trials [98]. CircRNA interventions have also been examined mainly at relatively early stages in preclinical models; for example, circRIMS2-associated synaptic and memory alterations were studied in four-month-old APP/PS1 mice [31]. Whether circRNA-directed interventions remain effective after substantial tau accumulation, synaptic loss, or neurodegeneration is unknown.

Future studies should evaluate circRNA interventions at different stages of AD, with established biomarkers used to define disease stage. Therapeutic effects should also be assessed using endpoints relevant to the proposed mechanism, including molecular targets, receptor function, synaptic plasticity, and cognitive outcomes. This will help determine whether circRNA manipulation produces effects that extend beyond molecular changes to functional improvement.

#### 7.6.5. Sex-Dependent Considerations for circRNA-Directed Therapy

Biological sex may influence circRNA dysregulation and response to circRNA-directed therapy. Female-specific downregulation of circHOMER1 has been reported in AD [4], suggesting that some circRNA alterations may differ between females and males. Whether these differences affect disease mechanisms or therapeutic response remains unknown.

Hormonal status may contribute to this variation. Oestrogen signalling has been associated with AD-related brain changes, tau pathology, neuroinflammation, and synaptic function [135,136,166], and oestradiol can also influence oxidative stress, iron homeostasis, and ferroptotic injury in experimental neural models [137]. However, no direct relationship between oestrogen signalling, circHOMER1, and ferroptosis has been established in AD. Future therapeutic studies should therefore include both sexes and, where relevant, consider menopausal or hormonal status when evaluating circRNA expression and treatment response.

#### 7.6.6. Experimental Models for Studying circRNA–Ferroptosis Interactions in AD

The proposed circRNA–ferroptosis relationships involve interactions among neurons, microglia, and astrocytes that are difficult to reproduce in monocultures or acute Aβ-exposure models. Human multicellular models may therefore help determine whether circRNA-dependent changes in iron homeostasis, lipid peroxidation, and glial responses are also observed when multiple neural cell types are present. Patient-derived cerebral organoids have been reported to develop Aβ-like aggregates together with increased lipid peroxidation and altered iron homeostasis, while ferrostatin-1 reduced these changes [141]. Incorporating iPSC-derived microglia, which exhibit graded inflammatory responses rather than discrete M1/M2 states [38], could further allow neuronal and glial ferroptosis-related processes to be examined within the same model.

Current cerebral organoids nevertheless have important limitations. Standard cerebral organoid protocols generally lack microglia, while astrocytes often remain immature and oligodendrocytes are limited or absent. These limitations are particularly relevant to ferroptosis because microglial iron metabolism, astrocytic antioxidant support, and oligodendrocyte-associated iron metabolism can influence neuronal susceptibility to lipid peroxidation. The extracellular environment also differs from that of the human brain, as organoids are commonly embedded in basement-membrane-rich matrices that do not reproduce the composition or mechanical properties of brain extracellular matrix. Models intended to study intercellular circRNA–ferroptosis interactions should therefore consider both cellular composition and the surrounding extracellular environment.

The absence of a functional vascular compartment creates an additional challenge. Increasing organoid size can generate hypoxic and necrotic regions, which may confound both ferroptosis-related measurements and circRNA biology. Cap-independent circRNA translation through IRES- and m6A-dependent mechanisms can increase under cellular stress, including hypoxia [94,96], while hypoxia itself is relevant to AD [97,98]. Non-vascularised organoids also cannot fully reproduce physiological delivery of transferrin-bound iron from the circulation. Vascularised or perfused models may therefore improve oxygen and iron delivery and reduce hypoxia within the model.

Developmental maturity should also be considered. Cerebral organoids generally resemble relatively early developmental stages, whereas AD develops in the ageing brain, where long-term changes in iron homeostasis, mitochondrial function, proteostasis, and antioxidant capacity may modify ferroptotic susceptibility. Extended culture or experimentally controlled ageing approaches may therefore improve disease relevance. Batch-to-batch and donor-to-donor variability should additionally be addressed through adequate biological replication and, where appropriate, isogenic controls.

Future models should integrate these features where possible. Microglia should be introduced at defined and reported stages; incorporation from approximately week 6, often within a week 6–8 window, may provide a useful framework for familial AD-oriented models, whereas later incorporation may be more appropriate for sporadic or later-stage disease models [167]. The timing of glial incorporation, maturation, vascularisation, and ageing procedures should be reported clearly because these variables can influence iron metabolism and cellular stress.

CircRNA analysis within these systems should also be cell-type specific. BSJ-specific quantification should identify which neural cell types express each candidate circRNA, and functional studies should then examine the circRNA in the relevant neuronal or glial population. These models could then determine whether circRNA-dependent effects identified in individual cell types are maintained in a human multicellular environment.

## Data Availability

No new data was generated or analysed in this study. Data sharing is not applicable to this article.

## Abbreviations

The following abbreviations are used in this manuscript:

AD	Alzheimer’s disease
Aβ	Amyloid-β
CircRNAs	Circular RNAs
ncRNAs	Non-coding RNAs
miRNA	microRNA
NF-κB	Nuclear Factor kappa-light-chain-enhancer of activated B cells
BACE1	Beta-site Amyloid precursor protein Cleaving Enzyme 1
APP	Amyloid Precursor Protein
NMDA	N-methyl-D-aspartate
UBE2K	Ubiquitin Conjugating Enzyme E2 K
GluN2B	Glutamate Ionotropic Receptor NMDA Type Subunit 2B
EPAC1	Exchange Protein directly Activated by cAMP 1
EPAC2	Exchange Protein directly Activated by cAMP 2
mRNA	Messenger RNA
RBPs	RNA-binding proteins
UBE2A	Ubiquitin-conjugating enzyme E2A
UCHL1	Ubiquitin C-terminal hydrolase L1
ER	Endoplasmic reticulum
A to I	Adenosine-to-inosine
ADARs	Adenosine deaminases acting on RNA
ATP13A1	ATPase class VI type 13A1
IL-1β	Interleukin 1 beta
TNF-α	Tumor necrosis factor alpha
IL-12	Interleukin 12
ROS	Reactive oxygen species
IL-10	Interleukin 10
CLIC1	Chloride intracellular channel protein 1
CSF	Cerebrospinal fluid
BSJ	Back-splice junction
QKI	Quaking
DHX9	DExH-box helicase 9
MBNL	Muscleblind-like protein
AMPA	α-amino-3-hydroxy-5-methyl-4-isoxazolepropionic acid
IRES	Internal ribosome entry sites
m6A	N6-methyladenosine
ADAM10	A disintegrin and metalloprotease 10
ADAM17	A disintegrin and metalloprotease 17
ceRNA	Competing endogenous RNA
TREM2	Triggering receptor expressed on myeloid cells 2
CDR-SB	Clinical Dementia Rating–Sum of Boxes
ARIA	Amyloid-related imaging abnormalities
PET	Positron emission tomography
PITRM1	Pitrilysin metallopeptidase 1
NOL10	Nuclear protein 10
TADA2A	Transcriptional adaptor 2A
BRAF	B-raf proto-oncogene, serine/threonine kinase
E2F1	E2 transcription factor 1
DHFR	Dihydrofolate reductase
GSE/GEO	Gene Expression Omnibus
UPR	Unfolded protein response
LTP	Long-term potentiation
Ca^2+^	Calcium
PKA	Protein kinase A
cAMP	Cyclic AMP
AKAP79	A-kinase anchoring protein 79
MAP1A	Microtubule-associated protein 1A
SIRT1	Sirtuin 1
TGF-β	Transforming growth factor β
IGF2R	Insulin-like growth factor 2 receptor
NiMA	Nutrient-induced Mitochondrial Activity
mTORC1	Mechanistic target of rapamycin complex 1
ZNF217	Zinc finger protein 217
TNFAIP1	Tumor necrosis factor, alpha induced protein 1
FGF7	Fibroblast growth factor 7
KIF1B	Kinesin family member 1B
KREMEN1	Kringle containing transmembrane protein 1
Smurf2	SMAD-specific E3 ubiquitin protein ligase 2
MAP2	Microtubule-associated protein 2
PSD95	Post-synaptic density protein 95
ddPCR	Droplet digital PCR
METTL3	Methyltransferase-like 3
RAPGEF4	Rap guanine nucleotide exchange factor 4
pAkt	Phosphorylated-Akt
RAPGEF3	Rap guanine nucleotide exchange factor 3
KEGG	Kyoto Encyclopedia of genes and genomes
Nrf2	Nuclear factor erythroid 2–related factor 2
4-HNE	4-hydroxynonenal
MDA	Malondialdehyde
iPSC	Induced pluripotent stem cell
TfR1	Transferrin receptor 1
STEAP3	Six-transmembrane epithelial antigen of the prostate 3
DMT1	Divalent metal transporter 1
LIP	Labile iron pool
NCOA4	Nuclear receptor coactivator 4
FPN1	Ferroportin 1
CDK5	Cyclin-dependent kinase 5
GSK-3β	Glycogen synthase kinase-3β
HO-1	Heme oxygenase-1
IRP/IRE	iron-regulatory protein/iron-responsive element
PUFA	polyunsaturated fatty acids
ACSL4	acyl-CoA synthetase long-chain family member 4
LPCAT3	lysophosphatidylcholine acyltransferase 3
AMPK/Sp1/ACSL4	Adenosine Monophosphate-Activated Protein Kinase/Specificity Protein 1
GPX4	Glutathione peroxidase 4
GSH	Glutathione
Nrf2	Nuclear factor erythroid 2-related factor 2
ARE	Antioxidant response element
FSP1	Ferroptosis Suppressor Protein 1
NAD(P)H	Nicotinamide Adenine Dinucleotide Phosphate
CoQ10	Coenzyme Q10
SLC7A11	Solute Carrier Family 7A11
SLC3A2	Solute Carrier Family 3A2
LRP8	low-density lipoprotein receptor-related protein 8
FTH1	ferritin heavy chain 1
QSM	Quantitative susceptibility mapping
SEC24B	SEC24 homolog B, COPII coat complex component
ALKBH5	AlkB homolog 5, RNA demethylase
